# Modulation of Functional EEG Networks by the NMDA Antagonist Nitrous Oxide

**DOI:** 10.1371/journal.pone.0056434

**Published:** 2013-02-14

**Authors:** Levin Kuhlmann, Brett L. Foster, David T. J. Liley

**Affiliations:** 1 Brain and Psychological Sciences Research Centre, Swinburne University of Technology, Hawthorn, Victoria, Australia; 2 Department of Neurology and Neurological Sciences, School of Medicine, Stanford University, Stanford, California , United States of America; French National Centre for Scientific Research, France

## Abstract

Parietal networks are hypothesised to play a central role in the cortical information synthesis that supports conscious experience and behavior. Significant reductions in parietal level functional connectivity have been shown to occur during general anesthesia with propofol and a range of other GABAergic general anesthetic agents. Using two analysis approaches (1) a graph theoretic analysis based on surrogate-corrected zero-lag correlations of scalp EEG, and (2) a global coherence analysis based on the EEG cross-spectrum, we reveal that sedation with the NMDA receptor antagonist nitrous oxide (N_2_O), an agent that has quite different electroencephalographic effects compared to the inductive general anesthetics, also causes significant alterations in parietal level functional networks, as well as changes in full brain and frontal level networks. A total of 20 subjects underwent N_2_O inhalation at either 20%, 40% or 60% peak N_2_O/O_2_ gas concentration levels. N_2_O-induced reductions in parietal network level functional connectivity (on the order of 50%) were exclusively detected by utilising a surface Laplacian derivation, suggesting that superficial, smaller spatial scale, cortical networks were most affected. In contrast reductions in frontal network functional connectivity were optimally discriminated using a common-reference derivation (reductions on the order of 10%), indicating that the NMDA antagonist N_2_O induces spatially coherent and widespread perturbations in frontal activity. Our findings not only give important weight to the idea of agent invariant final network changes underlying drug-induced reductions in consciousness, but also provide significant impetus for the application and development of multiscale functional analyses to systematically characterise the network level cortical effects of NMDA receptor related hypofunction. Future work at the source space level will be needed to verify the consistency between cortical network changes seen at the source level and those presented here at the EEG sensor space level.

## Introduction

It has been hypothesised that disruption of information integration within posterior parietal cortex is the agent invariant ‘final common pathway’ to drug-induced unconsciousness [1]–[3]. Although different drugs can have different modes of action at specific microscopic sites (i.e. facilitatory or suppressive) there may be common final network changes seen at a macroscopic level that underly drug-induced reductions in consciousness, whether they be in the form of a coma-like state as induced by general anesthetics or be it in the milder inattentive form of a dissociative and unresponsive state as induced by high doses of sedative drugs. To date the empirical evidence underpinning this hypothesis has exclusively involved inductive agents that: act principally through central 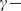 aminobutytic acid (GABA) agonism [1]–[5], manifest cerebral hypo-metabolism [6], [7] and are associated with the anteriorisation of slow wave EEG activity [8]–[10]. A vital test of this hypothesis would therefore involve the evaluation of the functional alterations in fronto-parietal networks induced by agents that do not fulfill these criteria. The dissociative anesthetic gas nitrous oxide (N_2_O, ‘laughing gas’) is an example of such an agent. N_2_O is believed to achieve its analgesic, sedative and hypnotic effects through the antagonism of N-methyl- D-aspartate (NMDA) receptor mediated activity [11]–[13], electroencephalographically it reduces frontal slow wave activity [14], [15], and metabolically brain activity either increases or remains unchanged during its administration [16], [17]. N_2_O and the other important NMDA receptor antagonist anesthetic, ketamine, are associated with psychoactivation, perceptual distortion, detachment from reality, and are therefore referred to as ‘dissociative’ agents [12], [13], [18]. Altered NMDA receptor function has been implicated in pharmacological models and treatments of a range of mental disorders such as schizophrenia [19], [20] and depression [21] respectively, thus elucidating the neurocognitive effects of ketamine and N_2_O may help better understand the neuropharmacological basis of these disorders.

We therefore sought to quantify changes in frontal, parietal and full brain networks obtained from high-density EEG during N_2_O inhalation using measures and methods that are capable of robustly assessing alterations in network topology and connection strength and, for the purposes of comparison, have been applied to GABAergic agents such as propofol. Such measures include global efficiency (GE) [3] and global coherence (GC) [22] calculated from multi-channel EEG recordings. GE is a time-domain graph theoretic approach and is essentially the average surrogate-corrected zero-lag cross-correlation over the EEG network. In contrast GC is a frequency domain measure and is defined here as the ratio of the largest eigenvalue over the sum of the eigenvalues of the complex EEG cross-spectral matrix. Increases and decreases in GE indicate increases and decreases, respectively, in global functional connectivity of the network considered. Similar properties for GC hold although one needs to take into account the eigenvector corresponding to the largest eigenvalue of the cross-spectral matrix (as outlined in the methods).

Analysing the GE and GC measures during N_2_O inhalation not only provides insight into the functional organisation of cortical networks, but if the measures change with the level of gas concentration and consciousness, then these measures could provide a means to monitor brain state and consciousness. It is also important to note that GE and GC have been applied by [3] and [22], respectively, to scalp EEG recordings of individuals undergoing anesthesia with the GABAergic antagonist propofol. For GE, propofol caused a breakdown in parietal network functional connectivity, whereas functional connectivity in the frontal network was relatively unchanged [3]. For GC, propofol caused full brain network decreases in GC at 11 Hz only during the transition to unconsciousness but not during unconsciousness. Moreover, globally coherent activity at 11 Hz shifted from a posterior predominance at rest to an anterior predominance during drug-induced unconsciousness [22]. Here we present the same methods applied to individuals undergoing sedation with the putative NMDA antagonist N_2_O in order to investigate the possibility of agent invariant final network changes that underly drug-induced reductions in consciousness.

To assess the spatial scales at which functional connectivity changes take place, GE and GC, were calculated using a simple multi-spatial scale analysis involving comparison of surface Laplacian (nearest neighbour; which passes high spatial frequencies) and common-reference (linked mastoids; which passes all spatial frequencies) derivations. For the case of propofol induction, Lee et al. [3] applied GE to a common-reference derivation, whereas Cimenser et al. [22] focused primarily on applying GC to a Laplacian-reference derivation. Here we consider both referencing schemes in the interests of a more complete understanding and comparison.

We show that increasing end-tidal N_2_O concentrations are associated with progressive alterations in full brain, parietal and frontal networks. In particular the Laplacian re-referenced GE method better detected full brain network changes and was required to observe any parietal network decreases in functional connectivity. On the other hand frontal decreases in functional connectivity were best detected with GE and GC using a common-reference derivation. Thus N_2_O, like the GABAergic agents propofol and desflurane, produces parietal level decreases in functional connectivity, which, based on our analysis, are dominated by local areal changes in superficial parietal networks.

## Materials and Methods

### Ethics Statement

This study was approved by the Swinburne University of Technology Human Research Ethics Committee.

### Experiment: Drug Administration, EEG and behavioural recordings

An analysis of a subset of the data described in the current manuscript, focusing on signal power changes, has been previously published [14], [23]. In the previous work, data corresponding to 20% and 40% inspired N_2_O were statistically analysed in detail, whereas only qualitative power changes for the 60% inspired N_2_O data were reported on [14]. The current analysis extends substantially the scope of this prior analysis by including the 60% N_2_O treatment group and developing an extensive functional connectivity analysis. As the experimental paradigm has been previously published [14], we recapitulate the key details here for clarity.

**Figure 1:**
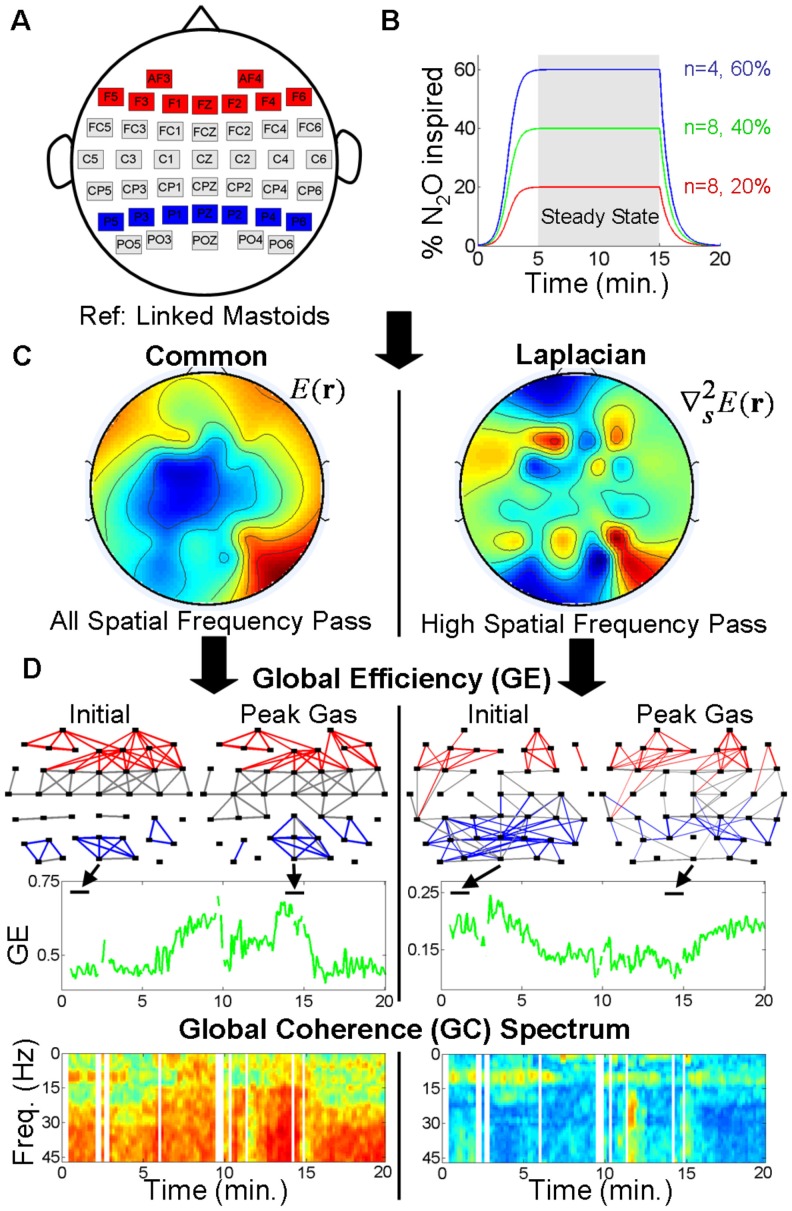
Experiment and analysis outline. (A) EEG was recorded with the extended 10–20 montage, then subsampled to 42 electrodes to directly compare common with Laplacian referenced data. Full, frontal and parietal networks were derived from all, red, and blue channels, respectively. (B) In addition to a 5 minutes eyes closed resting condition, participants underwent N_2_O gas inspiration over a 20 minute period with peak gas levels of 20, 40 or 60% (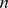 is the number of participants in each group). (C) EEG was recorded with a common linked mastoids reference sensitive to all spatial frequencies as seen in the potential map (left). EEG was also analysed with Laplacian re-referencing which is sensitive to high spatial frequencies (right). The potential maps correspond to snap shots of resting data and are presented just to illustrate the different spatial scales of the signals. (D) Global Efficiency (GE) and Global Coherence (GC) analyses were applied to common (left) and Laplacian (right) referenced data for full, frontal and parietal brain networks to assess global changes in functional connectivity resulting from N_2_O induction. Examples are given for a participant in the 60% peak gas group for the full brain network. For GE, network graphs are constructed based on surrogate-corrected zero-lag correlations. The graphs correspond to times of initial and peak gas inspiration. The thickness of the graph lines are proportional to the strength of the absolute value of the correlations which vary between 0 and 1. Only correlations of absolute magnitude greater than the 90th percentile value are shown. Red, blue and gray graph edges correspond to electrode pairs involving frontal, parietal, and neither frontal nor parietal electrodes, respectively. Note the decreases in parietal correlations with increased gas concentration for the Laplacian referenced derivation (right), consistent with the hypothesis of a ‘final common pathway’ to drug-induced reductions in consciousness. GE (green time series) for these weighted networks is the average of the absolute correlations. GC is the ratio of the largest eigenvalue over the sum of the eigenvalues of the complex cross-spectral matrix at each temporal frequency, this is reflected in the GC spectra (bottom). Vertical white spaces indicate time intervals in which data contained artefact. Additional measures were also derived from the GE and GC analyses to assess changes in functional connectivity.

Recordings of 62-channel EEG were obtained from twenty human subjects during the inhalation of N_2_O to fixed steady-state end-tidal concentrations. During EEG recording, responsiveness was also monitored using an auditory continuous performance task (aCPT). Figure 1 provides an outline of the experiment and analysis.

Healthy males (general medical examination passed, no neurological or psychiatric history) were recruited through written informed voluntary consent and females were excluded due to greater risk of nausea and emesis with N_2_O. Before the recording session, each participant was randomly allocated to 1 of 3 conditions: 20%, 40%, or 60% inspired N_2_O/O_2_, respectively. For conditions 1 to 3 there were 8, 8 and 4 participants, respectively. Fewer participants were recruited for the 60% gas condition due to problems with nausea and emesis. Participants fasted for 8 hours before the recording which commenced at 9am. Baseline involved a 5-minute eyes-closed recording of spontaneous EEG during the aCPT. Recordings of EEG during N_2_O inhalation were 20 minutes in duration which also involved the aCPT. This period comprised a 5-minute equilibration phase, a 10-minute period of continuous equilibrated gas flow at the end of which N_2_O was discontinued with 100% O_2_ administered for a further 5 minutes during a washout phase. N_2_O and O_2_ were administered through a closed 1.5-m Bain coaxial non-rebreathing circuit. Using a standard clinical pulse oximeter finger clip, O_2_ saturation and heart rate were obtained. End-tidal concentrations of N_2_O, O_2_ and CO_2_ were determined online using a Normocap (Datex-Ohmeda, GE Healthcare, Madison, WI) infrared gas analyzer. Every minute the outputs from both the pulse oximeter and gas analyzer were manually logged. EEG recordings were performed in a noise-minimized laboratory using a 62-channel Syn-Amps EEG system (NeuroScan; Compumedics Ltd., Melbourne, Australia). The EEG montage was positioned according to the extended international 10∶20 system, with a linked mastoids reference. The sampling rate was 500 Hz and digital hardware bandpass filtered the signal between 0.1–70 Hz. The aCPT used to monitor behavioural state required the participants to respond (left or right button) to 2 auditory tones of differing frequency (1 or 2 kHz, respectively), but of fixed stereo amplitude (70 dB) occurring every 2.5 seconds with a slight jitter during baseline and gas recordings. Targets and responses (accuracy and latency) were automatically logged for each trial.

### EEG Analysis

Artifact-rejected raw 62-channel EEG was further bandpass filtered between 1 and 40 Hz. Any remaining artifact such as eye blinks and electromyograph were successfully removed using independent-component analysis (ICA) as implemented in the EEGLAB toolbox [24]. Additional artifacts that could not be removed with ICA were clipped from the data with continuity assumed across segments separated by clipped data. Analysis involved common referenced or Laplacian re-referenced data in order to characterise brain network dynamics at essentially two different spatial scales. By using a surface Laplacian referencing scheme, in which each electrode is re-referenced with respect to an average computed from nearby electrodes, activity can be weighted towards local, radially oriented, cortical (superficial) sources [25]. The surface Laplacian of the raw signals 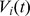 is computed as:
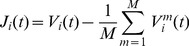
(1)where 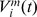 indicates the voltage recorded at one of the 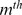 closest electrodes to electrode 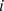. The choice of 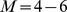 depended on the electrode location and the symmetry of nearby electrodes and follows a previously described high-density Laplacian derivation [22]. Applying the Laplacian re-referencing reduced the number of channels to 42, with marginal channels being excluded due to an insufficient number of neighbouring electrodes. The same 42 channels were analysed for common-reference and Laplacian-reference derivations. Two global network analyses were considered: (1) GE, (2) GC. Full brain, frontal and parietal networks were studied with each method. The full brain network involved all 42 channels. The frontal network involved 9 channels: AF3, AF4, FZ, F1-F6. The parietal network involved 7 channels: PZ, P1-P6. GE provides a graph theoretic approach to quantifying the transfer of information between nodes of a network by multiple parallel paths [26]. This approach relies on the creation of networks represented by undirected weighted graphs where, in the case of EEG, weights are defined as the surrogate-corrected genuine zero-lag cross-correlations [3]. GE is then calculated as the average of the inverse shortest path length over all pairs of nodes in the network, i.e. the average surrogate-corrected zero-lag cross-correlation over the network. GE can also be calculated on unweighted and fully-connected networks in order to dissociate the contributions of network topology and connection strength to observed alterations in GE. In contrast to the time-domain approach used for the calculation of GE, GC quantifies network dynamics based on computing the cross-spectrum over a range of spectral frequencies as a function of time [22]. GC summarises information in this cross-spectral matrix and is typically defined as either the ratio of the largest eigenvalue of the matrix to the sum of the eigenvalues [27], or as the average of all pairwise coherences [28]. Here we use the former approach as it was used by Cimenser et al. [22] in application to the study of propofol. Despite the differences in their calculation, which we describe in detail below, GE and GC provide complementary perspectives on a more general correlation structure, the cross-correlation function. The cross-correlation function which describes correlations with different time lags can be obtained by the inverse Fourier transform of the cross-spectrum. GE views only zero-lag correlations from the cross-correlation function, whereas GC at a specific frequency (i.e. 11 Hz) is dependent on the power of the corresponding frequency component of the cross-correlation function.

In our analysis we consider GE and GC measures as functions of N_2_O gas concentration, aCPT accuracy and reaction time in order to assess the changes induced by N_2_O inhalation. This is done by generating GE- and GC-based time series using specific sliding windows. The empirically obtained time-series of N_2_O gas concentration, aCPT accuracy and reaction time are interpolated to the start times of GE and GC windows in order to histogram data and generate the desired functions presented in the results. Specifically, gas concentration and aCPT reaction time were linearly interpolated, whereas aCPT accuracy was interpolated using the nearest neighbours.

#### Statistical Assessment

To statistically assess the differences in the measures across rest and different gas concentration histogram bins, one-way analysis of variance (ANOVA) was applied to each measure data set. Multi-comparison tests were also applied to statistically assess the difference between the mean measure for the rest data and the mean measure for any given data-bin of interest. Significance levels of 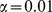 and 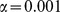 with Bonferroni correction for multiple comparisons were considered. Moreover, because one-way ANOVAs gave very high significance (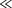
0.001) in a large number of cases, a non-parametric receiver operator characteristic (ROC) analysis [29] was applied to quantify the separability of the different gas concentration bin distributions from the rest distribution. A ROC analysis is useful for the purposes of devising accurate monitoring methods where one wants to know how separate the data distributions are rather than just how different the data means are. If one can separate the distributions well, then high classification accuracy of the monitoring methods can be achieved. The area under the ROC curve (AUROC) provides a measure of the separability of two distributions that depends on both sensitivity and specificity, with areas close to 1 meaning high separability and an area of 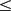
0.5 indicating chance, or worse, performance. A rough guide to classifying such separability using the AUROC is: 0.9–1.0 = excellent classification, 0.8–0.9 = good classification, 0.7–0.8 = fair classification, 0.6–0.7 = poor classification and 0.5–0.6 = failure [30]. Appendix S1 for an explanation of the ROC analysis employed here). One-way ANOVAs, multi-comparison tests and ROC analysis were also applied to statistically assess the differences in the measures across rest and different aCPT accuracy bins, and also across rest and different aCPT reaction time bins. In order to visualise the ROC analyses, bar charts of the AUROC scores are presented, where AUROC score bars point up or down if the difference in the corresponding median measure value relative to rest reflects an increase or decrease, respectively. If the difference is zero then the AUROC is multiplied by 0 and this is reflected in the bar chart. In the cases where the difference is zero AUROC scores are likely to be below chance. This was true for all such cases where the the difference was zero in the results. In cases where a given bin had less than 10 samples AUROC values were not computed because a small number of samples is inadequate to characterise the data distribution. This is also reflected in the AUROC bar chart by the absence of bars.

### Global Efficiency (GE) Analysis

Our GE implementation follows the approach of Lee et al. [3] and is reiterated here for clarity. The data were broken into 12 second segments with an 8 second overlap. Each segment was then split into 4 second sub-segments with 3.6 seconds of overlap giving a total of 21 sub-segments. These 21 segments represent the original data ensemble. For each sub-segment 50 Fourier-phase randomised surrogates were generated [31], producing a 50×21 surrogate data ensemble for a 12 second window. For each segment, the Pearson's equal-time cross correlation coefficient was calculated across channels for each sub-segment of the original data ensemble and the surrogate data ensemble, using the following definition:
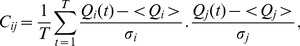
(2)where 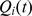 corresponds to either the raw common-referenced data, 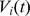, or the Laplacian-referenced data, 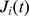. A genuine correlation matrix, 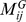, which corrects the total correlations by taking into account the random correlations of the surrogate data [32], [33] was calculated as:
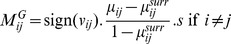
(3)where 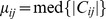, 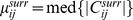 and 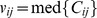 (‘med’ indicates median over the sub-segments of the ensemble). Note that self connections (i.e. 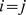) were ignored. The factor 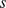 in equation (3) evaluates the significance of the difference 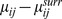. Since a Gaussian distribution of 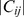 and 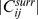 cannot be guaranteed, the Mann-Whitney-Wilcoxon U-test was used with the null hypothesis of equal medians. If it was rejected, then 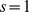; otherwise, 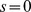. The p-value was set as 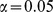 and the Bonferroni correction for multiple U-tests for M-dimensional matrix elements was applied: 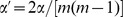 where 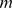 is the number of channels. For each full brain, frontal and parietal network, weighted, unweighted and all-to-all networks were constructed. Within a given 12 second segment, the genuine correlation matrix, 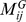, was used to create ‘genuine’ weighted networks and un-weighted networks. In a weighted network, labelled 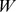, the weight between electrodes 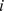 and 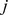 is defined by 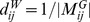, which is treated as the shortest path length between the two electrodes, and as mentioned above, self connections are ignored. In an un-weighted network, labelled 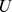, the shortest path lengths between nodes are 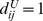 for non-zero valued 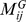 and 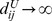 for zero valued 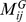. In an all-to-all network, labelled 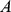, all connections are weighted by 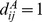. By comparing the weighted and the un-weighted networks one can gain a sense of the effects of connection strengths on the network measures to be analysed. Measures of global efficiency were calculated for weighted, un-weighted and all-to-all networks. Global efficiency quantifies the efficiency of information transmission of a network based on the average weight of edges that must be traversed to go from one node to another [26]. The global efficiency of a network was determined by:
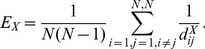
(4)where 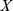 corresponds to network 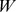, 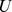 or 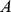, and 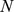 is the number of channels. 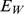 and 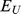 take on values between 0 and 1, while 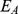 is 1. If 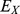 is small then the information transmission is inefficient and correlations are low, whereas if 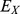 is close to 1 then information transmission is efficient and many channels are correlated. The contribution of connection strength to global efficiency was defined as the ratio between the global efficiencies of the weighted network and the un-weighted network:
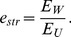
(5)The contribution of network structure to global efficiency was defined as the ratio between the global efficiencies of the un-weighted network and the all-to-all network:
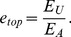
(6)It can be noted that 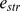 and 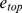 are between 0 and 1.

### Global Coherence (GC) Analysis

Our GC implementation follows the approach of Cimenser et al. [22] and is reiterated here for clarity. The data was broken into 0.5 second non-overlapping sub-segments. Adjacent sub-segments were clustered into segments of 40 sub-segments per segment (20 seconds) and segments had 50% overlap. Each sub-segment was detrended by removing the best straight line fit (using CHRONUX [34] software):
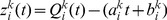
(7)where 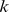 indexes each sub-segment and 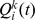 corresponds to the 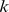
th sub-segment of either the raw common-referenced data, 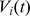, or the Laplacian-referenced data, 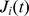. For each sub-segment the tapered 512-point FFT of all channels was windowed with the first Slepian sequence [35], 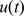, which is normalised such that 
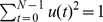
. The FFT is given by:
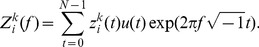
(8)Within a segment, the corresponding sub-segment FFTs were mean corrected:
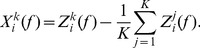
(9)The average power spectrum for all channels was then calculated:
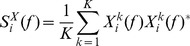
(10)where 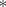 indicates the complex conjugate. From this the average cross spectrum for all channel pairs was calculated:
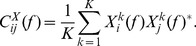
(11)The eigenvalues 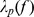 and eigenvectors 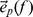 of the cross spectrum, 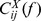, for each frequency were then computed [36]. Eigenvalues were sorted in increasing order, eigenvectors were sorted accordingly. It can be noted that the eigenvectors are normalised according to 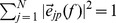. The variables 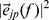, which are termed the row weights, represent the contribution of the 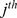 electrode to the 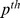 eigenvector and therefore provide a description of which electrodes contribute to significant and coherent activity when the 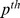 eigenvalue is large.

Within a trial, the global coherence for each frequency was computed as
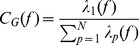
(12)where 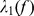 is the largest eigenvalue and 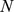 is the number of eigenvalues (i.e. number of channels). For a given frequency, if 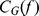 is small then the cross-spectral power is likely to be weak and uncorrelated across channels, whereas if 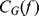 is close to 1 and the magnitude is high across many ‘channel’ elements in the eigenvector/row weights corresponding to the largest eigenvalue, then these channels with high magnitude are considered coordinated. In the current manuscript we focus on the 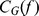 values, however, some consideration is given to the eigenvectors in Appendix S1.

**Figure 2:**
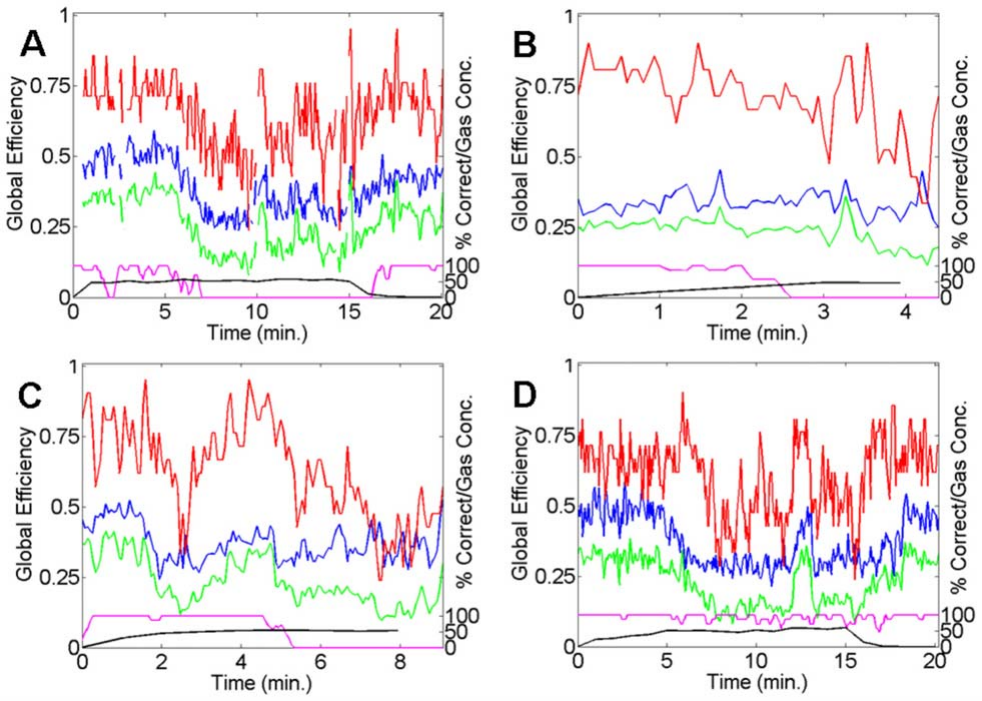
Time series of the GE-based functional connectivity measures for the four different subjects (A–D) from the 60% peak gas group for the parietal networks obtained with Laplacian re-referencing. In each sub-figure: GE (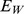; green), the contribution of connection strength to GE (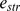; blue), and the contribution of network topology to GE (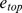; red). Black curve: measured end-tidal N_2_O gas concentrations. Magenta curve: smoothed auditory task performance. Missing data indicates artefact.

**Figure 3:**
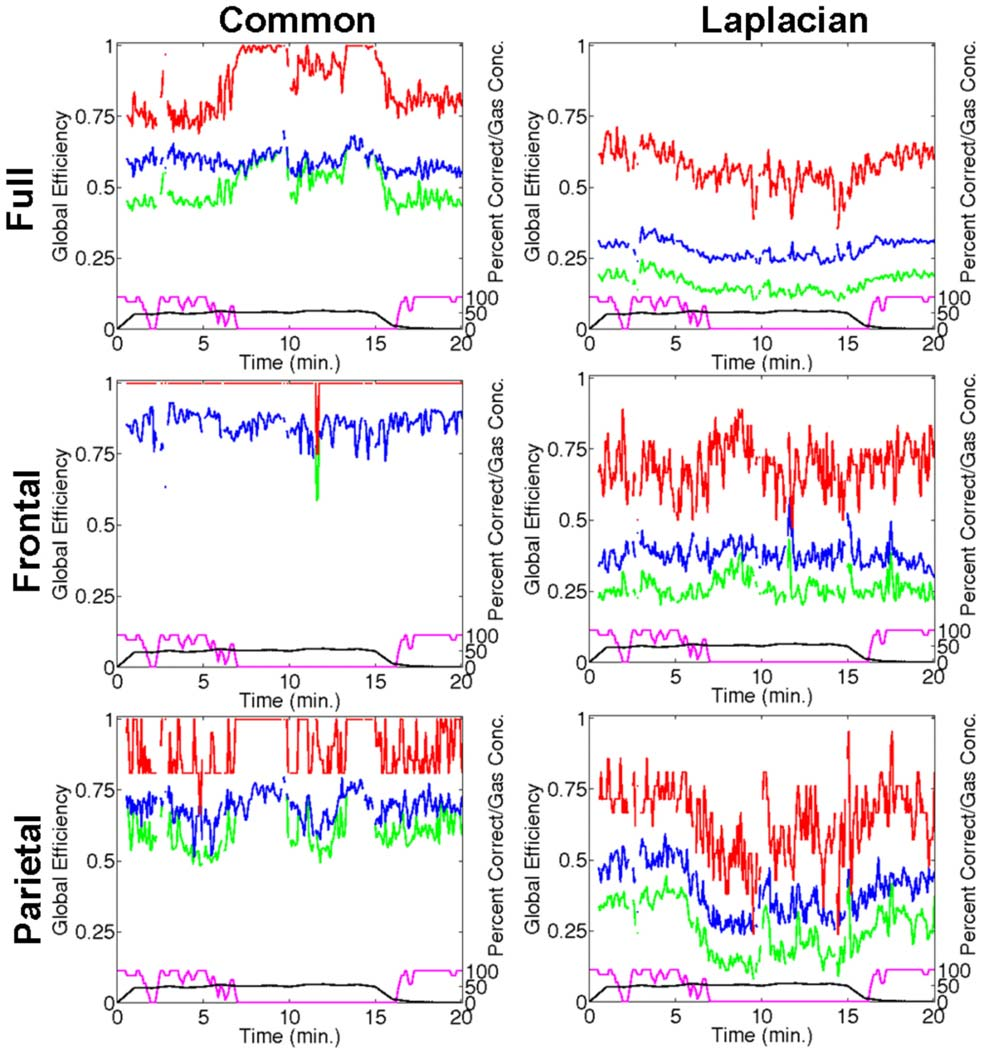
Time series of the GE-based functional connectivity measures for the same subject in **Figure 1D** from the 60% peak gas group for the full brain (top row), frontal (middle row) and parietal (bottom row) networks obtained either with common-reference (left column) or Laplacian re-referencing (right column). In each sub-figure: GE (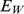; green), the contribution of connection strength to GE (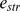; blue), and the contribution of network topology to GE (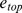; red). Black curve: measured end-tidal N_2_O gas concentrations. Magenta curve: smoothed auditory task performance. The bottom right sub-figure corresponds to Figure 2A. Missing data indicates artefact.

The GC spectra were computed for frequencies up to 47 Hz, however, the majority of the analysis presented here focuses on GC in the 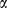 band around 11 Hz. This is because strong resting 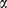 band power was a common feature across all participants, and there were intermittent decreases in 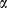 band power, especially for high gas concentrations (Example time-frequency power spectra are given in Figures 2 and 3 in Appendix S1). This frequency of 11 Hz was also the frequency focused on in a similar study involving propofol [22] due to its strong GC.

## Results

As expected the changes in brain state under the influence of N_2_O were best observed for high gas concentrations. First we broadly summarise our results in terms of the spatial scales and the networks at which the significant changes take place. Then we expand on specifics in the following sections.

For GE-based functional connectivity, it was found that GE measures (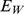, 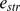, 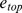) generally decreased for the parietal network with a Laplacian-reference derivation when N_2_O gas concentration increased to 60% and responsiveness decreased. This indicates that decreases in parietal network functional connectivity occur on a smaller scale as consciousness is reduced. Moreover, the GE measures generally decreased for the frontal network with either reference derivation indicating frontal decreases in functional connectivity may occur on all scales as consciousness is reduced. The GE measures increased and decreased for the full network with the common-reference and Laplacian-reference derivations, respectively, although changes were most significant for the Laplacian-reference derivation. This indicates that full brain decreases in functional connectivity are most likely to occur at smaller scales when consciousness is reduced.

Increases in gas concentration and reductions in responsiveness were also linked to decreases in GC-based functional connectivity at 11 Hz for the frontal network with a common-reference derivation; and decreases for the full network with the Laplacian-reference derivation. These decreases in GC-based functional connectivity were largely consistent with the GE-based changes for the same network and scale conditions. Considering the GE- and GC-based frontal network changes together, decreases in functional connectivity may predominantly occur on on larger scale as consciousness is reduced.

In the following sections we address specific details of spatial scale and network dependent changes in functional connectivity. First we present the most signficant finding of the study, the individual results for the 60% peak gas group showing decreases in GE-based functional connectivity for the parietal network with the Laplacian-reference derivation. This is consistent with GE-based functional connectivity changes seen for propofol [3], and can therefore be considered as evidence to support the hypothesis that breakdown in functional connectivity in parietal networks reflects an agent invariant final network change underlying drug-induced reductions in consciousness. Then, we give examples of the GE- and GC-based changes for an individual from the 60% peak gas group. We note the results of an analysis of the dependence of the measures on gas concentration, aCPT accuracy and reaction time for of all of the subjects from the 20, 40 and 60% peak gas groups combined. Given that the measures considered were largely independent of subject responsiveness for the subjects in the 20 and 40% peak gas groups, additional analysis of the 60% peak gas group alone is presented and the dependence of the measures on gas concentration and responsiveness is illustrated.

### Individual GE-based parietal network changes

Figure 2 presents the GE (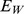, 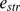 and 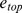) time series for the parietal network with a Laplacian-reference derivation for the individuals of the 60% group (end-tidal gas equilibration between 5 and 15 minutes). This figure demonstrates that for each subject for the parietal network with a Laplacian-reference derivation, decreases in GE-based functional connectivity were observed during high gas levels and loss of responsiveness (zero-level performance in the auditory task). This provides strong support for the parietal network being consistently involved in agent invariant final network changes underlying drug-induced reductions in consciousness. For the individuals' data presented in Figure 2A, B, C and D, the AUROC scores for the difference between rest and ‘incorrect’ data are 0.87, 0.36, 0.95, and 0.86, respectively. Where ‘incorrect’ implies the subject either gave an incorrect response, which was extremely rare during rest, or they did not respond at all during a given time window of the auditory task. These scores are highly significant and show parietal GE decreases occur during loss of responsiveness, except for the subject in Figure 2B. Although one can see a slight decrease in the GE measures towards the end of the recording for the subject in Figure 2B, this subject's recording did not reach the peak gas equilibration period due to emesis and therefore significant GE changes were not observed. The subject in Figure 2C also experienced emesis and their recording lasted less than 10 minutes, however, this was long enough for equilibrated gas levels to be reached after 5 minutes and for significant parietal network changes to be observed.

### Spatial scale and network dependence: Individual example

For the same subject in Figures 1D and 2A from the 60% case, Figure 3 shows the time series of the GE measures for the full brain (top row), frontal (middle row) and parietal (bottom row) networks obtained either with common-reference (left column) or Laplacian re-referencing (right column). For this subject for the full brain network during the equilibrated 60% gas period, the GE measures either showed increases or decreases depending on the respective use of a common-reference or Laplacian-reference derivation. For this subject these changes were strongly correlated with the loss of responsiveness. These GE time series indicate that the efficiency of information transmission is spatial scale dependent. For the data presented in Figure 3 the significant AUROC scores for the difference between rest and ‘incorrect’ data for the 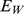 measure were 0.91, 0.92, 0.88 and 0.87, for the full network with common-reference, full network with Laplacian-reference, frontal network with Laplacian-reference, and parietal network with Laplacian-reference, respectively. This indicates clearly indicates that these 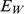 changes are linked to unresponsiveness for this individual.

For Laplacian re-referencing the smaller (and more superficial) spatial scales are associated with reduced transmission/correlations for all networks, whereas the common-reference derivation, which conflates all spatial scales, is associated with increases in transmission/correlations in the full brain network. In the common-reference derivation, correlations would be in part due to volume conduction effects. Baseline differences in the GE measures seen just prior to N_2_O inhalation (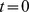) for common-reference and Laplacian derivations should therefore give an indication of the likely influence of volume conduction effects. From Figure 3 it can be further noted that for this subject strong frontal network changes were not observed and that large parietal changes are only seen with Laplacian re-referencing indicating that parietal network changes linked to conscious state may operate on a local scale in posterior parietal cortex when there is a significant loss of response.

**Figure 4:**
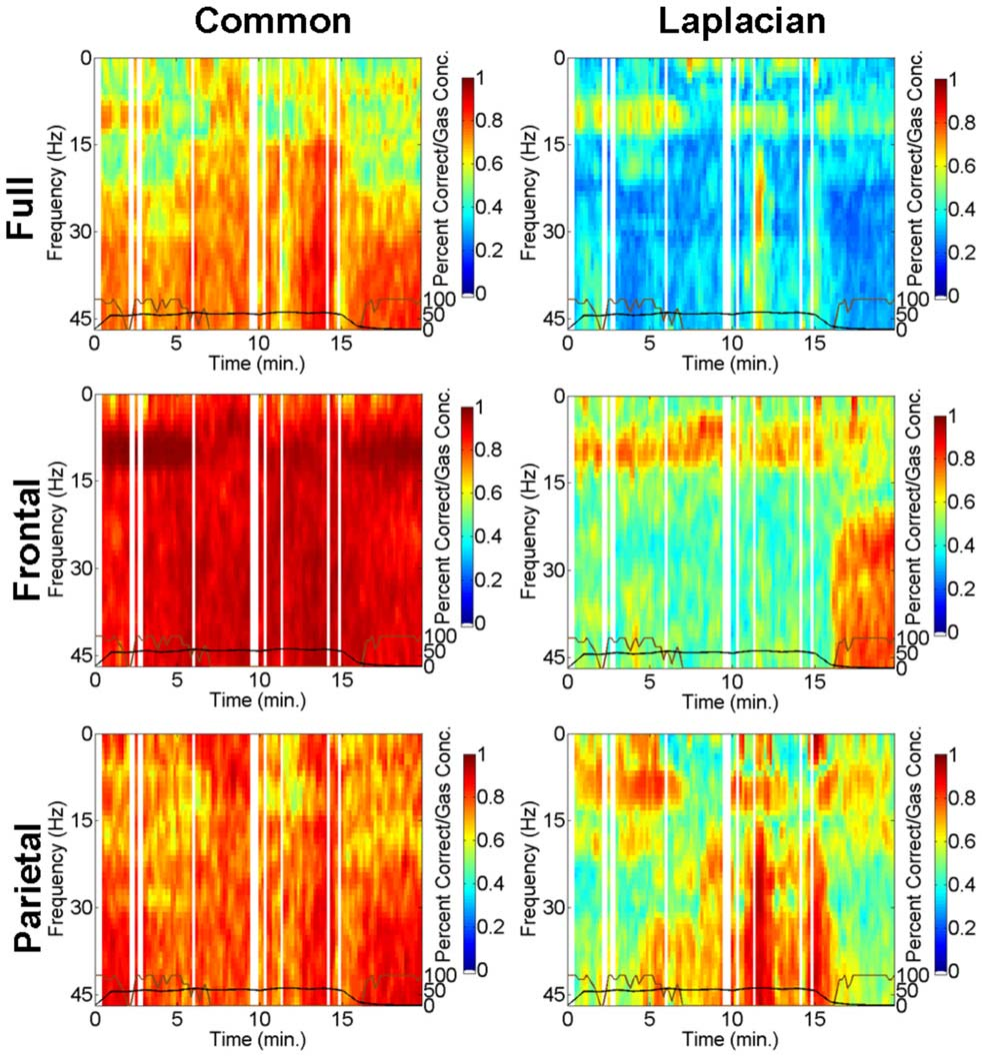
GC-based functional connectivity spectra for the same subject in **Figure 3** for the full brain (top row), frontal (middle row) and parietal (bottom row) networks obtained either with common-reference (left column) or Laplacian re-referencing (right column). In each sub-figure: Black curve - measured end-tidal N_2_O gas concentrations; brown curve - smoothed auditory task performance. Vertical white spaces indicate time intervals in which data contained artefact.

**Figure 5:**
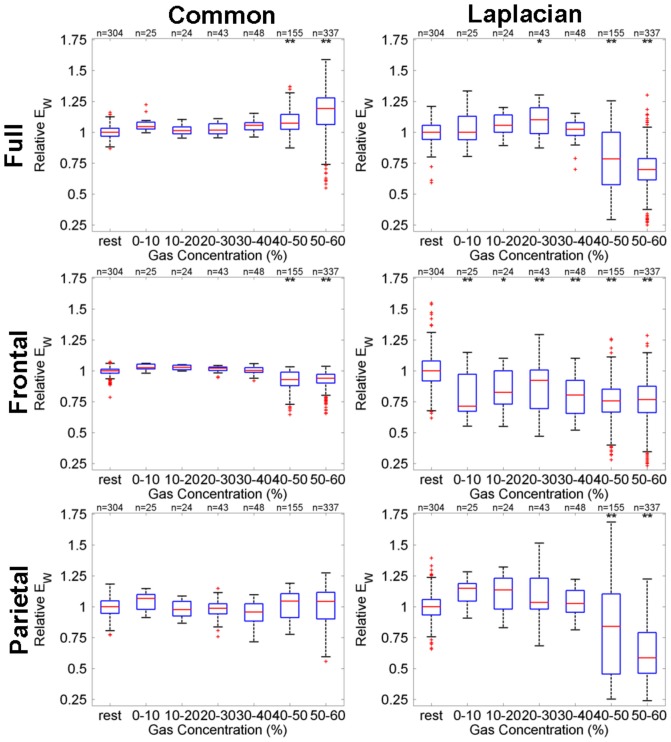
Dependence, for the 60% peak gas group, of GE-based functional connectivity (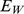) defined relative to the median during rest on N_2_O gas concentration for full brain (top row), frontal (middle row) and parietal (bottom row) networks, obtained either with common-reference (left column) or Laplacian re-referencing (right column). Box-whisker details: Red horizontal lines indicate bin distribution median; Upper and lower blue box edges correspond to 75th and 25th percentiles, respectively; Black dashed whiskers span 99.3% of the distribution assuming a normal distribution; The red crosses indicate outliers. The bins labelled above by 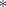 and 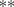 indicate the mean is statistically significant from rest for significance levels of 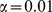 and 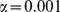 corrected for multiple comparisons, respectively.

As was shown in Figure 2 these parietal level changes in GE were a common feature in all 60% peak gas subjects, however, frontal changes were also observed across the 60% peak gas group. Similar, though less pronounced, changes were seen with GC. Example data from the GC analysis is presented in Figure 4. For the same 60% peak gas subject as in Figure 3, Figure 4 illustrates GC spectra (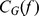) for the full brain (top row), frontal (middle row) and parietal (bottom row) networks obtained either with common-reference (left column) or Laplacian re-referencing (right column). GC is seen strong across all frequencies for the common-reference derivation, highlighting volume conduction and common-reference effects, whereas Laplacian re-referencing gives prominence to GC in the 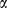 band around 11 Hz. Moreover, Figure 4 shows that applying the Laplacian derivation to full brain and parietal level networks reveals that 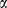 band (around 11 Hz) GC-based functional connectivity is intermittently weakened during peak gas effect, however such changes were not as prominent as the changes seen for the GE measures. Nevertheless, changes in GC when going from rest to gas inhalation appear to be related to a slight anterior shift in coherent activity in the full brain network as is observed for the same subject by considering snap shots of the row weights corresponding to the largest eigenvalue at 11 Hz (see Figure 4 and Figure 5 in Appendix S1), as well as a cumulative row weight analysis (see Figures 20 and 21 and Tables 1–3 in Appendix S1). For the data presented in Figure 4 the significant AUROC scores for the difference between rest and ‘incorrect’ data for the 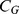 at 11 Hz measure were 0.69, 0.80, 0.73, 0.66, and 0.70 for the full network with common-reference, frontal network with common-reference, parietal network with common-reference, full network with Laplacian-reference and frontal network with Laplacian-reference, respectively. This clearly indicates that these 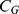 at 11 Hz changes are linked to unresponsiveness for this individual, but less strongly than the corresponding GE measures.

### Spatial scale and network dependence: Group analysis

To assess the sensitivity of all the measures to N_2_O gas concentration, aCPT accuracy or reaction time, data were pooled across subjects. Pooling, as opposed to an individualised analysis, seemed most appropriate because not all subjects received the same N_2_O gas dosage so we could not compare peak gas cases within individuals. Before pooling, each measure was defined as relative to the median value of the measure during the rest recording simply by dividing by the median rest value. This was done to correct for the intersubject variability in the resting state measure values and in some sense it de-individualises the data. Moreover, while all rest data were considered in the analysis, only the first 15 minutes of the gas recordings for each subject were included, as there are strong power rebound effects that occur upon N_2_O withdrawal [14].

#### Combined group data dependence on gas concentration and responsiveness

Although the combined 20%, 40% and 60% group data showed a strong dependence of the measures on gas concentration (Figures 7–Figures 9 in Appendix S1), the combined group data showed little dependence of the measures on responsiveness (Figures 10–12 in Appendix S1 for aCPT accuracy and Figures 13–15 in Appendix S1 for aCPT responsiveness). Therefore here we focus on the 60% peak gas group data below. The combined group data dependence of the measures on high gas concentrations, was quite similar to that seen for the 60% peak gas group.

#### 60% peak gas group data dependence on gas concentration

Figure 5 shows box-whisker plots for GE-based functional connectivity (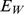) defined relative to the median during rest for the full brain (top row), frontal (middle row) and parietal (bottom row) networks obtained either with common-reference (left column) or Laplacian re-referencing (right column). It can be seen that across the 60% peak gas group, full brain GE increases using a common-reference with increasing gas concentration, but decreases when using Laplacian re-referencing. For frontal networks both referencing schemes show a reduction in GE with increasing gas concentrations, whereas for the parietal networks a decrease in GE for high gas concentrations is only seen when a Laplacian-reference derivation is used.

**Figure 6:**
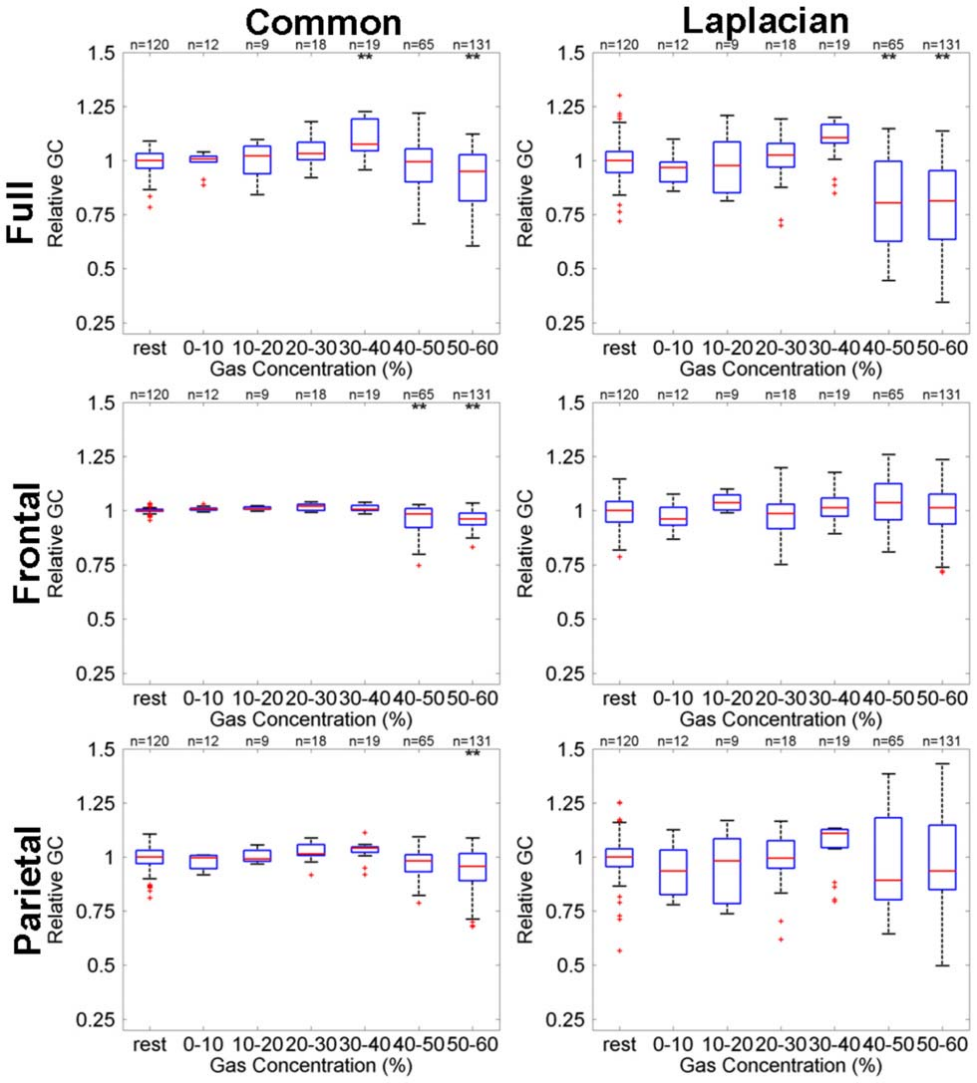
Dependence, for the 60% peak gas group, of GC-based functional connectivity (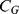) at 11 Hz defined relative to the median during rest on N_2_O gas concentration for full brain (top row), frontal (middle row) and parietal (bottom row) networks, obtained either with common-reference (left column) or Laplacian re-referencing (right column). Box-whisker and multi-comparison test significance marker (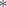, 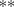) details are the same as for Figure 5.

For comparison, Figure 6 shows box-whisker plots for GC-based functional connectivity (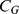) at 11 Hz defined relative to the median during rest for the full brain (top row), frontal (middle row) and parietal (bottom row) networks obtained either with common-reference (left column) or Laplacian re-referencing (right column). It can be seen that there are clear full brain decreases in GC, for both derivations and high gas concentrations. Frontal network GC decreases are restricted to common-reference derivations. Given the strong overlap in the GC distributions for the different bins it is clear that GE is more sensitive to changes in brain networks than GC at 11 Hz.

**Figure 7:**
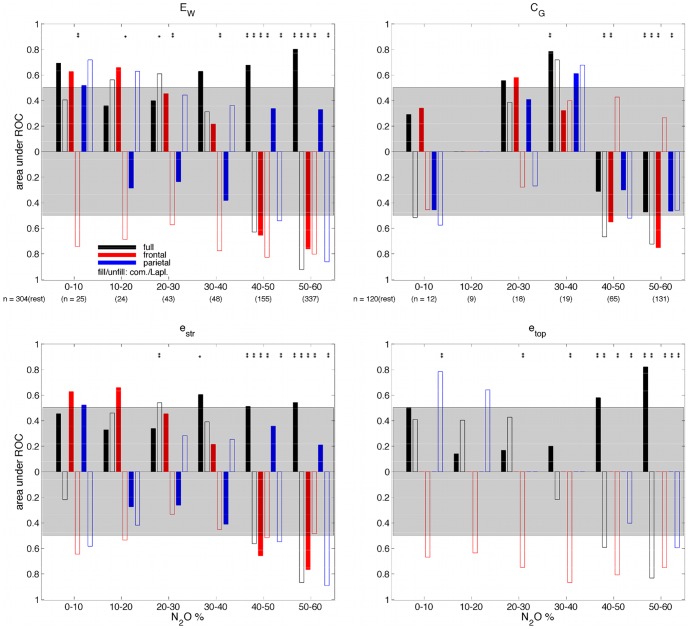
AUROC as a function of inspired N_2_O concentration for the 60% peak gas group for (top left) weighted global efficiency, 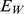, (top right) global coherence, 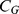, at 11 Hz, (bottom left) the contribution of connection strength to global efficiency, 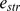, and (bottom right) the contribution of connection topology to global efficiency, 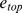. One-way ANOVAs were typically significant across rest and the six gas concentration bins for each of the measures (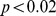 for the GE measures, 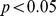 for the GC measure except for parietal networks with Laplacian-reference derivation). The difference in the median measure value relative to rest is indicated by the the direction of the respective bar (up, increase; down, decrease). Gray shading indicates ROC curve areas 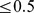 signifying a chance or worse ability to discriminate between randomly chosen gas and rest measures. Thus AUROC 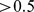 for a given measure implies a better than chance likelihood of being able to discriminate between a gas state and rest. Multi-comparison test significance marker (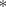, 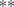) details are the same as for Figure 5.

Figure 7 summarises the results of the ROC analysis for all GE and GC measures as a function of gas concentration for the 60% peak gas group, where AUROC score bars point up or down if the difference in the corresponding median measure value relative to rest reflects an increase or decrease, respectively. Bars stretching beyond the gray region indicate greater than chance separability. GE measures are generally found to provide better separability of the high gas concentration data and the rest data than the GC measures. Above chance separability from rest is seen in the 40–50% and 50–60% gas concentration bins, with the most significant separability seen for the GE measures for the 50–60% gas concentration bin. In the top left of Figure 7, for GE (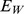) evaluated over all electrodes using the Laplacian derivation, the decreases in 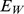 seen with higher gas concentrations (Figure 5) are better correlated with gas level than the increases in 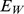 seen using the common-reference derivation. This suggests that the dominant N_2_O induced changes involve decreases in functional connectivity in local superficial cortical networks. For GE (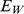) evaluated over a frontal subset of electrodes the decreases in 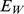 observed with higher gas concentration (Figure 5) for both referencing schemes appear to be equally well correlated with gas levels based on the AUROC scores.

For GE (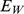) evaluated over the parietal subnetwork using the Laplacian derivation the decreases in 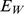 observed with higher gas concentration (Figure 5) are strongly dependent on gas level. No parietal level network changes are seen using a common-reference derivation. In the bottom left and bottom right of Figure 7 similar trends are observed with the connection-strength-based and topology-based GE measures 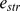 and 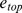, respectively, except for frontal 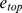 which continually showed high 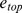 values largely because the unweighted frontal network was almost always fully connected. Full brain network GC (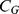) values calculated at 11 Hz (top right of Figure 7), at high inspired N_2_O levels, are only meaningfully different from baseline value using a Laplacian reference derivation, whereas frontal brain network GC values are best detected using a common-reference derivation. Considering the GE and GC measures together full brain and parietal network changes are best seen using a Laplacian-reference derivation, whereas frontal network changes are best observed using a common-reference derivation.

The bins which were determined to have statistically significantly different means from the rest bin (indicated by 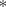 and 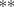 for significance levels of 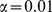 and 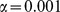 corrected for multiple comparisons, respectively) were largely consistent with the above chance AUROC results for high gas concentrations.

#### 60% peak gas group data dependence on responsiveness

**Figure 8:**
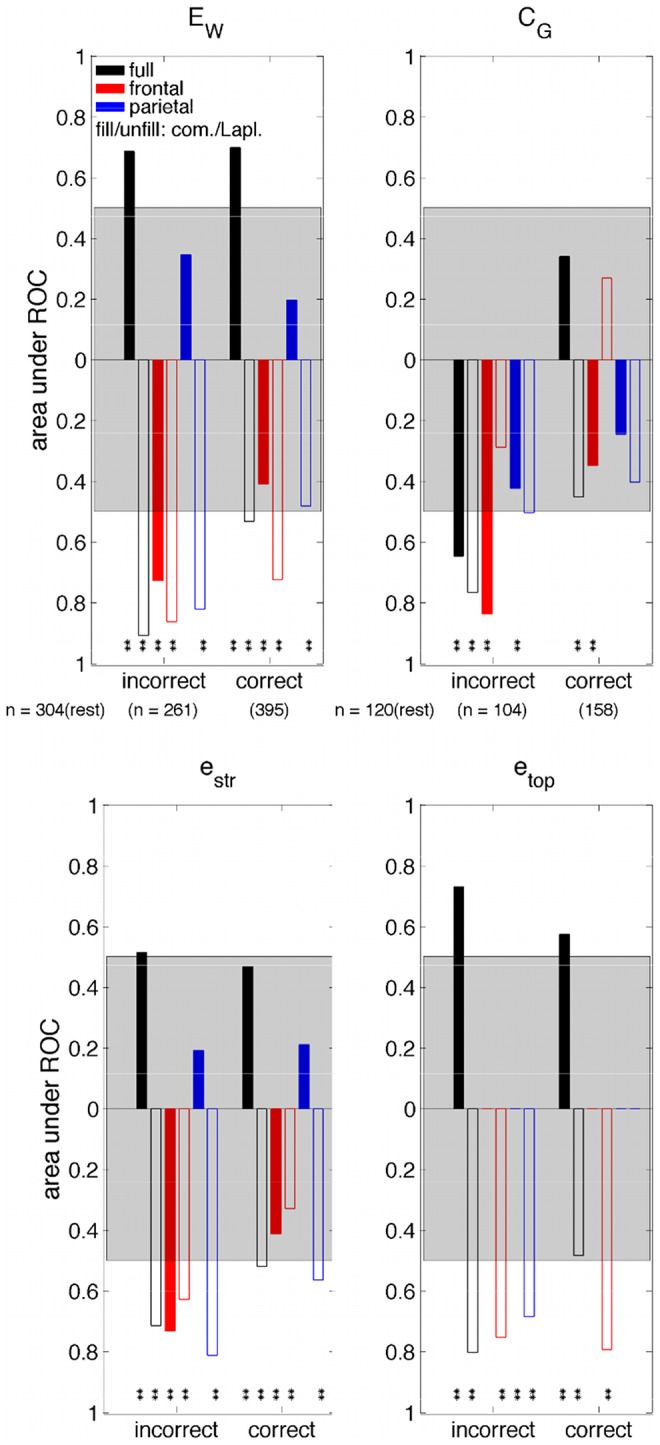
AUROC as a function of aCPT accuracy for the 60% peak gas group for (top left) weighted global efficiency, 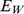, (top right) global coherence, 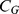, at 11 Hz, (bottom left) the contribution of connection strength to global efficiency, 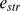, and (bottom right) the contribution of connection topology to global efficiency, 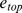. One-way ANOVAs were typically significant across rest and the two accuracy bins for each of the measures (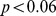 for the GE measures except for parietal 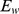 and 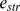 with common-reference derivation, 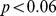 for the GC measure except for the parietal network with Laplacian-reference derivation). The difference in the median measure value relative to rest is indicated by the the direction of the respective bar (up, increase; down, decrease). Multi-comparison test significance marker (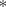, 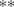) details are the same as for Figure 5. The remaining features are the same as described in Figure 7.

**Figure 9:**
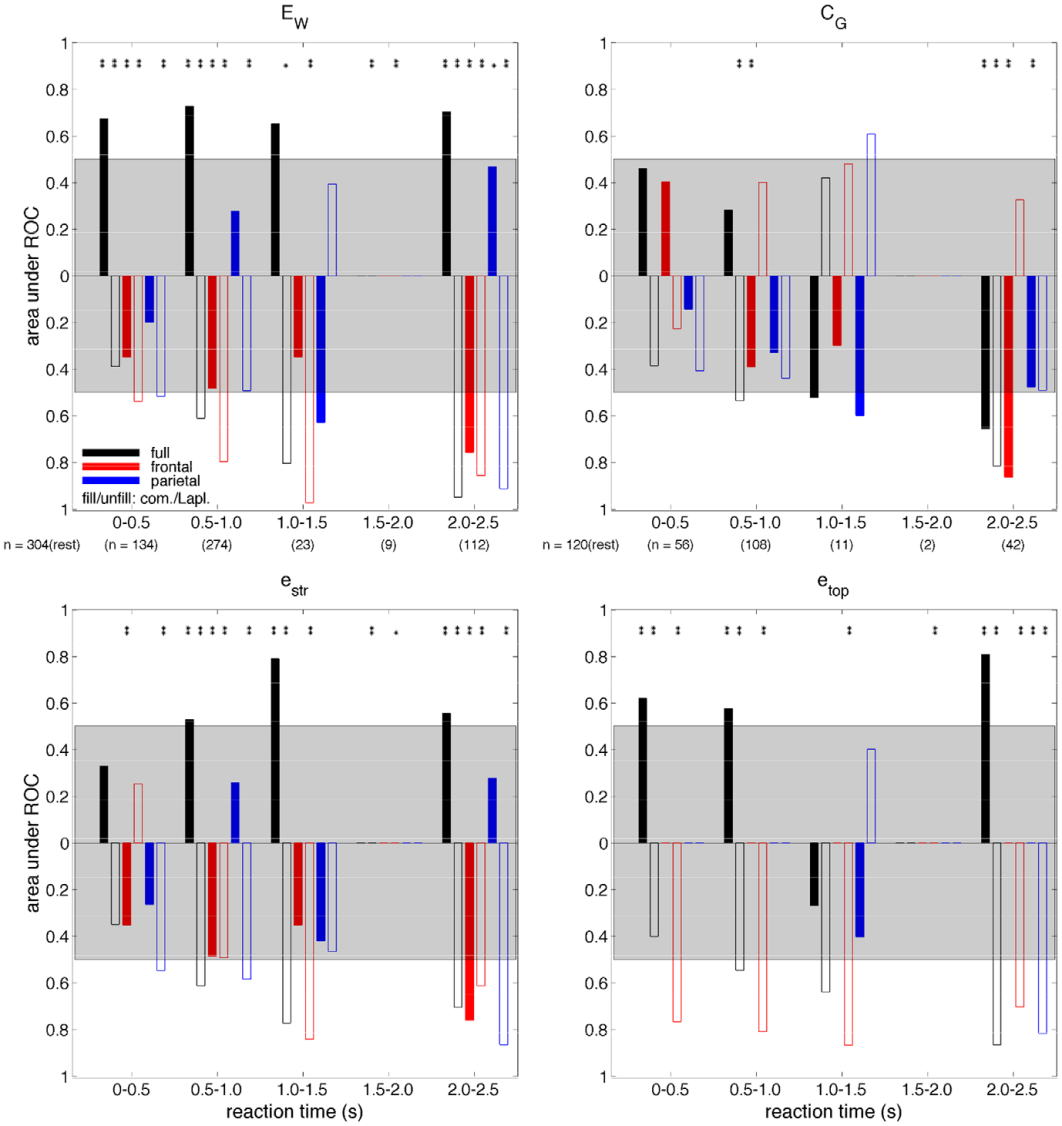
AUROC as a function of aCPT reaction time for the 60% peak gas group for (top left) weighted global efficiency, 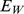, (top right) global coherence, 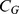, at 11 Hz, (bottom left) the contribution of connection strength to global efficiency, 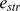, and (bottom right) the contribution of connection topology to global efficiency, 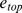. One-way ANOVAs were typically significant across rest and the five reaction time bins for each of the measures (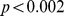 for the GE measures except for frontal 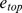 with common-reference derivation, 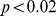 for the GC measure). The difference in the median measure value relative to rest is indicated by the the direction of the respective bar (up, increase; down, decrease). Multi-comparison test significance marker (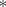, 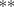) details are the same as for Figure 5. The remaining features are the same as described in Figure 7.

The ROC-based dependence of the measures on auditory task responsiveness for the 60% peak gas group are presented in Figures 8 and 9 as a function of aCPT accuracy, and aCPT reaction time, respectively. In Figure 8, the ‘correct’ and ‘incorrect’ bins correspond to data collected from the gas recording. Moreover, ‘correct’ implies the subject answered correctly, whereas ‘incorrect’ implies the subject either gave an incorrect response (which was extremely rare during rest) or they did not respond at all. The significant changes are similar to those observed for the gas concentration analysis, namely reductions in responsiveness (i.e. reductions in task accuracy reflected in the ‘incorrect’ bin in Figure 8 and increases in task reaction times reflected in the ‘2–2.5 s’ bin in Figure 9) were linked to 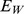 decreases for the parietal network with a Laplacian-reference derivation; 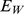 decreases for the frontal network with either reference derivation; and 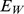 increases and decreases for the full network with the common-reference and Laplacian-reference derivations, respectively. Reductions in responsiveness were also linked to 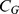 decreases for the frontal network with a common-reference derivation; and 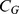 decreases for the full network with either reference derivation. The box-whisker plots showing the dependence of GE (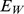) and GC (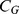) on aCPT accuracy for the 60% peak gas group are presented in Figures 16 and 17 in Appendix S1, respectively. While the box-whisker plots showing the dependence of GE (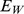) and GC (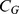) on aCPT reaction time for the 60% peak gas group are presented in Figures 18 and 19 in Appendix S1, respectively.

Although here we have analysed measure dependence on gas concentration, aCPT accuracy and aCPT reaction time independently, increases with gas concentration were found to be correlated with decreases in responsiveness (i.e. decreases in aCPT accuracy and increases in aCPT reaction time), as is shown in Figure 6 in Appendix S1.

### GE analysis with sub-sampled 10–20 montage

The results for the GE study with propofol were obtained using 21 channels in a 10–20 montage [3]. We found that if we re-performed our analysis by subsampling our electrodes to create a 10–20 montage, similar results to those we observed with 42 channels were obtained, as presented in Figures 22 and 23 in Appendix S1.

## Discussion

Because a range of structurally unrelated compounds were able to induce anesthesia it was initially speculated that they all acted through a common mechanism. The Meyer-Overton hypothesis gave form to such speculations by asserting that the empirically observed relationship between anesthetic potency and lipid solubility was a reflection of a progressive non-specific disruption of neuronal membrane function [37]. However because such a unitary hypothesis of action failed to account for a number of well established empirical phenomena [38] it was ultimately abandoned. Anesthetics are now known to exert their differential effects by interacting with a diverse range of microscopic level cellular and molecular targets [12]. Nevertheless, as our results show, sedative levels of the dissociative agent N_2_O produces GE-related decreases in parietal network functional connectivity that are also reported for the structurally unrelated inductive anesthetic agents propofol [3] and sevoflurane [39], thus giving weight to the view that macroscopically anesthetics may be altering the state of consciousness through unitary network level mechanisms. Other network level analyses, utilising resting state functional MRI (rs-fMRI) [4], [40] and EEG [3], [8], [41] with a range of anesthetic agents, are consistent with these selective alterations in parietal activity. Most significantly rs-fMRI studies involving midazolam [40], sevoflurane [39] and propofol [4], [5] reveal reductions in default mode network activity that are typically accompanied by changes in fronto-parietal functional connectivity. The relationship between the above changes and increases in frequency-specific effective connectivity between anterior and posterior cingulate cortex during propofol induction [42] still needs to be understood in greater detail and the consistency of changes across studies needs to be thoroughly evaluated.

By applying the global functional connectivity measures GE and GC to high density EEG recorded during N_2_O inhalation we found changes in full brain, parietal and frontal level cortical networks. We probed the scale of these changes using common and surface Laplacian reference derivations. A surface Laplacian provides a spatial filtering of the scalp EEG that limits electrode sensitivity to more local and superficial sources, thereby revealing source dynamics at smaller spatial scales than common-reference scalp potential derivations [25]. The Laplacian also has the advantage of minimizing the electromyographic (EMG) contamination of EEG [43], important given the demonstrated effects of well known anesthetic agents on resting muscle tone [44].

While full brain network changes were best observed using a Laplacian-reference derivation, frontal decreases in functional connectivity were best observed using a common-reference derivation suggesting that activity over a range of spatial scales is perturbed by the action of N_2_O. In contrast parietal network decreases in functional connectivity could only be detected as reductions in GE using a Laplacian reference derivation, indicating that only the finer spatial structure of superficial parietal networks is likely affected by N_2_O.

### Differences in dependence on responsiveness

To understand the behavioural results it is worthwhile to note several points. On a first inspection it was thought that behavioural changes were not very significant when the 20, 40 and 60% peak gas groups were combined because there were incomplete gas recordings for 2 of the 4 subjects of the 60% peak gas group. However, as is shown in Figures 8 and 9 significant behavioural changes are seen in the 60% peak gas group. Instead, the main reason for the discrepancy seems to be that for some of the subjects in the 20 and 40% peak gas groups there were several drops in task accuracy involving up to a few minutes of unresponsiveness. For reasons not yet fully understood, these changes in responsiveness were generally not found to coincide with significant changes in our measures for subjects who experienced equilibrated gas concentrations below 40%. We can note from Figure 7 (and also Figure 9 in Appendix S1) that most significant measure changes relative to rest occurred at gas concentrations above 40% (although based on visual inspection in some individual cases there are clear changes in the measures when responsiveness drops). Therefore there are likely to be brain changes occurring at concentrations below 40% that cause brief unresponsiveness with the measures we have considered here not generally sensitive to these changes. This may be because the unresponsiveness occurring at different gas concentrations and/or different rates of inhalation is linked to different kinds of network dynamics. Alternatively, and in addition, it is possible that the distracting dissociative effects at low gas concentrations were sufficient to interrupt task performance. Further consideration of changes in responsiveness for equilibrated gas concentrations below 40% is beyond the scope of this study and will be a subject of future investigation.

### Comparison to GE and GC studies of propofol

Strong decreases in GE have also been reported in parietal networks during propofol anesthesia [3]. However these authors evaluated GE using only 21 channels and a common reference, and thus our results are not directly comparable. Because they found changes with a common-reference, it may be that propofol and N_2_O affect parietal level activity at different spatial scales, however a re-analysis with GE of the existing propofol data using a Laplacian-reference derivation will need to be performed to fairly evaluate the basis for such a speculation. Given that Lee et al. [3] evaluated GE using only 21 channels, we recalculated GE and GC during N_2_O inhalation for the standard 10∶20 montage by subsampling and found that this did not qualitatively alter our results, regardless of whether a common-reference or Laplacian-reference was used (see Figures 22 and 23 in Appendix S1).

The ability of GE to discern parietal network changes reflects the general ability of a network theoretic approach to better detect N_2_O induced changes compared to the frequency domain correlational approach of GC. Nevertheless our GC analysis did have one important advantage in that a direct comparison to propofol with a Laplacian-reference derivation could be made. During propofol anesthesia it is found that global coherence in the 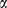 band does not disappear during loss of consciousness, but intermittently disappears during transitions from consciousness to unconsciousness [22]. We observed similar effects with N_2_O (Figure 4) for the GC spectrum of the full brain network with the Laplacian. As end-tidal N_2_O gas concentration increased to 60% the alpha rhythm intermittently disappears which may be a reflection of the brain transitioning towards unconsciousness as was the case for propofol. As such, high concentrations of N_2_O may predominantly induce a ‘transitional state’ on the way to unconsciousness, rather than induce complete unconsciousness. Such a transitional state would reflect reductions in consciousness, where the degree of the reduction in consciousness depends in part on the gas level, the individual and prior exposure to similar substances. Very high concentrations of N_2_O may induce complete unconsciousness, however, this is difficult to study given the attendent problems of nausea, emesis and hypoxia.

Propofol also induces an anteriorisation of global coherence at 11 Hz during loss of consciousness as shown with a cumulative row weight analysis [22]. We observed a similar, though smaller, anterior shift during high N_2_O gas concentrations (see Appendix S1, Tables 1–3 and Figures 4, 5, 20 and 21 in Appendix S1 for details) and this weaker shift may be indicative of a ‘transitional state’. In addition, it was found that cumulative univariate power, as an alternative to cumulative row weight, plots (see Figure 20 in Appendix S1) can capture the same shifts and thus brings into question the additional utility of the more complicated cumulative row weight analysis for the purposes of monitoring brain state during anesthesia [22]. Although the row weight approach is related directly to correlations and is therefore a network approach, on a first inspection with N_2_O-based data it appears it could be strongly biased by univariate power measures. A more thorough comparison would be required to make a final judgement.

### EEG spectral content during N_2_O inhalation

In the current paper our GC analysis focused primarily on a frequency of 11 Hz as we saw large power and GC changes in this band, especially at 60% N_2_O gas concentrations. In prior studies, Rampil et al. [45] found that compared to rest there were frontal increases in power in the theta, beta, 40–50 Hz and 70–110 Hz bands at 50% N_2_O gas concentrations, whereas Yamamura et al. [15] found an increase in power around 34 Hz and a decrease in power in the 8–13 Hz bands at 50–70% N_2_O gas concentrations. More recently for a subset of the data presented here, Foster and Liley [14] found that there were frontal decreases in power in the 1–4 Hz and the 4–8 Hz bands while the 8–15 Hz alpha band saw minimal change in relative power, although absolute power decreases did occur, at 40% N_2_O gas concentrations. If one considers the 60% peak gas group as we do here, consistent with the results of Yamamura et al. [15] one finds there are strong intermittent drops in the alpha band power as can be seen for a given subject in the GC spectra in Figure 4 and the time-frequency power spectra (see Figures 2 and 3 in Appendix S1). These changes were consistent across subjects in the 60% peak gas group (not shown here). It is important to note the data analysed here and in Foster and Liley [14] was collected in a noise-minimized non-clinical environment, 1–40 Hz band-pass filtering was applied and independent components analysis was used to remove artefact. These are the likely reasons we do not see increases in theta and high frequency power under N_2_O inhalation as observed by Rampil [45]. Rampil used a single frontal bipolar montage and thus the strategies available for artefact rejection were much restricted.

### True correlations and reductions in consciousness

The present study suffered from an important limitation that future studies will need to address. Unlike previous studies evaluating network level changes that attend anesthesia, most participants in our study did not completely lose consciousness in the sense observed for standard general anesthetics. Operationally, loss of consciousness is assessed by loss of response to noxious stimuli. The subjects in the 60% peak gas group did lose responsiveness, in terms of task performance, for significant periods of time, therefore from an operational perspective these subjects experienced some reduction of consciousness. Although more standard probes for responsivness would have better characterized patient state, such techniques can interfere with EEG signals, and are not completely commensurate with unconsciousness [46].

The reason the majority of the subjects in the study did not lose consciousness is because N_2_O is an anesthetic gas of relatively weak potency. It is estimated that only fifty percent of people will lose responsiveness at an endtidal N_2_O concentration of 63% (MAC awake) [47]. In contrast the potent halogenated agent sevoflurane has a MAC awake of 0.4–0.6% [48]. As mentioned above, at N_2_O concentrations near 63% the incidence of nausea and vomiting is high thus precluding easy empirical scrutiny of the effects of N_2_O on brain activity. Nevertheless we found that in the participants who did lose responsiveness that parietal network functional connectivity decreases preceeded or attended unresponsiveness.

It is also worthwhile noting that our surface Laplacian was evaluated using a simple nearest-neighbour method which may have poorly estimated radial current densities due to not adequately taking into account the geometry and electrical properties of the skull and scalp. More accurate estimates could be determined by analytically calculating the surface Laplacian from spherical spline interpolations of densely sampled (64 or 128 channel) EEG data [25], [49] or by using realistic, MRI derived, head models [50]. However such extra effort may go unrewarded as nearest-neighbour and spherical spline Laplacians have been found empirically found to give essentially equivalent topographic and chronometric information [51]. Moreover, although working in source space, as opposed to sensor space as we have done here, may put one ‘closer’ to the true cortical networks and help alleviate potential confounding correlations resulting from field effects, the results one obtains in the sensor space are still dependent on the source reconstruction approach applied [52]. Therefore we choose to take the iterative approach of seeing what one obtains at the sensor level and comparing this to other studies done with other drugs, such as propofol, at the sensor level using the exact same methods [3], [22], before considering future studies at the source level. Moreover, the application of techniques at the sensor level, bypasses the extra computational complexity of source reconstruction, allowing for the potential application of the measures considered to real-time monitoring of individuals undergoing drug-induced sedation or anesthesia.

Given we have chosen to work first at the sensor level it is important to point out possible confounds. One is that computation of cross-spectral/coherency (or correlation) measures using a common-reference derivation is confounded by power and phase changes in the common-reference signal [53]. Under certain assumptions this can be avoided by considering the imaginary part of the coherency [54], however, here we wished to reuse the methods of GE [3] and GC [22] used for propofol. Since Lee et al. [3] focused on a common-reference and Cimenser et al. [22] focused on a Laplacian-reference we decided to perform our analysis on both reference derivations. When working in sensor space, the confounds due to the common-recording reference can be reduced in part by Laplacian re-referencing, but true reference-free recordings cannot be obtained from the EEG, and can only be obtained from magnetoencephalography (MEG). To assess if the GE and GC changes observed here at the sensor level are truly observed in cortical networks, future studies with EEG and MEG will need to be done at the source level. Nevertheless, given that a Laplacian-reference derivation is less confounded by a common-recording reference and that here for the 60% peak gas group we have observed parietal decreases in GE for the Laplacian-reference derivation during N_2_O inhalation, we can be quite confident that these changes are occurring at the level of cortical networks.

It is also important to emphasise that although a common-reference can confound correlations, making it more difficult to be certain if the correlations we observe are true cortical correlations or just sensor level correlations, the confounds are consistent. This means that if the measures are shown to be dependent on gas concentration and responsiveness at the sensor level, as is done here, then these measures can still potentially be used in monitoring devices for sedation and anesthesia.

Another possible confound is random correlations due to noise or the finite size data effect [33]. Here the GE measure is surrogate-corrected (as mentioned in the methods) in order to remove such confounds, as was the case in Lee et al. [3] in their GE analysis of propofol. Surrogate-correction was not applied here to the GC analysis as this was not performed by Cimenser et al. [22] in their GC analysis of propofol, and we sought to provide a direct comparison with N_2_O. The lack of surrogate-correction for GC may be one potential explanation for why the GC measure was generally less sensitive to changes in N_2_O gas concentration when compared to the GE measures.

## Conclusions

Similar to propofol, the dissociative and weak anesthetic agent N_2_O reduces functional connectivity in the scalp EEG most strongly in the parietal area. This effect seems to specifically occur as the subjects experience reductions in consciousness; and provides strong evidence that completely different families of anesthetic drugs reduce consciousness via a common mechanism of parietal network disruption. Moreover, our results demonstrate that N_2_O perturbs functional connectivity over a number of spatial scales, thus opening up the possibility that this may be a key feature of anesthetic action. Future studies will therefore need to employ a variety of other imaging modalities, such as rs-fMRI and MEG, to characterise what scale dependent effects are shared by anesthetic agents having differing pharmacological modes of action.

## Supporting Information

Appendix S1 **Additional Methods and Results.** Summarises ROC analysis and additional GC methods. Provides additional power spectral, GC results as well as GE and GC results for the combined group, the 60% peak gas group and for a 10–20 montage. The relationship observed between gas concentration and responsiveness is also provided.
(PDF)
