# Supplementary material for: Modulation of Functional EEG Networks by the NMDA Antagonist Nitrous Oxide
Source: PLoS One. 2013 Feb 14;8(2):e56434. doi: 10.1371/journal.pone.0056434 (PMC3572968; doi:10.1371/journal.pone.0056434)
Supplement: Appendix S1 — Additional Methods and Results. Summarises ROC analysis and additional GC methods. Provides additional power spectral, GC results as well as GE and GC results for the combined group, the 60% peak gas group and for a 10–20 montage. The relationship observed between gas concentration and responsiveness is also provided. (PDF) [file pone.0056434.s001.pdf]

Appendix S1: Additional Methods and Results

Supplementary Information for

Modulation of functional cortical networks by the NMDA antagonist nitrous oxide

Levin Kuhlmann[1], Brett L. Foster[2], David T.J. Liley[1].

1. Brain and Psychological Sciences Research Centre, Swinburne University of Technology, Hawthorn, Victoria, Australia.
2. Department of Neurology and Neurological Sciences, School of Medicine, Stanford University, Stanford, California, United States.

Contents

1 Methods **1**
1.1 Additional Global Coherence and Power Analysis . . . 1
1.2 ROC analysis . . . 2

2 Results **3**
2.1 Example Time-Frequency Power Spectra . . . 3
2.2 Row Weight Snap Shots . . . 4
2.3 Relationship Between Gas Concentration and Responsiveness . . . 5
2.4 Combined 20%, 40% and 60% Peak Gas Group Results . . . 6
2.4.1 Combined group data dependence on gas concentration . . . 6
2.4.2 Combined group data dependence on responsiveness . . . 6
2.5 60% Peak Gas Group Box Whisker Plots for Behavioural Data . . . 18
2.6 Additional Global Coherence and Power Analysis . . . 18
2.7 Analysis involving subsampling down to 10:20 Montage . . . 19

1 Methods

1.1 Additional Global Coherence and Power Analysis

Additional measures quantifying anteriorisation and posteriorisation of globally coherent activity or power were considered. These measures of anteriorisation/posteriorisation, called directed row weight sum (dRWS) and directed power sum (dPS), were defined as:

$$\mathrm{dRWS}(f) = \sum_{j=1}^{N} y_j |\vec{e}_{jp}(f)|^2 \tag{1}$$

and

$$\mathrm{dPS}(f) = \sum_{j=1}^{N} y_j S_j^X(f) \tag{2}$$

where y_j is the co-ordinate value of the jth electrode along the nose-occiput axis, with Cz corresponding to $y = 0$ and frontal and posterior positions being positive and negative, respectively. The y values were normalised relative to the maximum distance of the electrodes from Cz. Positive and negative values of dRWS and dPS indicate anteriorisation and posteriorisation, respectively. Power was considered in addition to the row weights, because it is not based on the cross-spectrum and therefore acts as a control against cross-spectral changes. For the group analyses, rather than define these directional measure changes relative to the median for the rest data by dividing by the median of the rest, the median of the rest was instead subtracted out. This makes it easier to assess anteriorisation/posteriorisation changes.

1.2 ROC analysis

A ROC analysis was used to non-parametrically assess the separability of the different measure distributions from the rest measure distribution, grouped depending on different gas concentrations, auditory task performances, or reaction times as described in the manuscript. Here we describe the ROC analysis and illustrate how the area under a ROC curve (AUROC) is generated from the comparison of a pair of measure distributions. A ROC analysis involves a binary classification of two distributions using a varying discriminating threshold.

In the top of Figure 1 one can see two normal distributions, A (left; red) and B (right; magenta), with different means and the same standard deviation ($\mu_A = -1$, $\sigma_A = 1$; $\mu_B = 1$, $\sigma_B = 1$). The black line is the discrimination threshold which is shifted along the measure axis to perform classification across all possible threshold values. When the threshold is at a given position on the measure axis, measure values falling to the left of the threshold are classified as belonging to distribution A and are called 'negatives', whereas measure values falling to the right of the threshold are classified as belonging to distribution B and are called 'positives'. Note that the ROC analysis is symmetric and therefore it does not matter which distribution is called positive or negative, each distribution just needs a different label. The positives classified correctly and falsely are referred to as 'true positives' (TP) and 'false positives' (FP), respectively. The negatives classified correctly and falsely are referred to as 'true negatives' (TN) and 'false negatives' (FN), respectively. In the top plot of 1 the proportions of TPs, FPs, TNs, and FNs correspond to the areas of the light blue, dark blue, light green, and green regions, respectively. It can be noted that: the proportion of TPs is the area between the two distribution curves and to the right of the threshold; the proportion of FPs is the area below distribution A and to the right of the threshold; the proportion of TNs is the area between the two distribution curves and to the left of the threshold; and the proportion of FNs is the area below distribution B and to the left of the threshold. The sensitivity is then defined as

$$S = \frac{TP}{TP + FN} \tag{3}$$

i.e. the proportion of correct positives among all positives sampled, where TP and FN represent the proportions of TPs and FNs, respectively. The false positive rate is then defined as

$$FPR = \frac{FP}{FP + TN} \tag{4}$$

i.e. the proportion of incorrect positives among all negative samples, where FP and TN represent the proportions of FPs and TNs, respectively.

By varying the discrimination threshold one can obtain sensitivity and false positive rate estimates for the full range of possible thresholds. This data forms the basis of the ROC curve, shown in the bottom of Figure 1, which plots sensitivity versus false positive rate (i.e. $1 -$ specificity). The black square shows the point corresponding to where the threshold is positioned in the top part of Figure 1. Perfect classification/separability corresponds to a sensitivity of 1 and a false positive rate of 0, therefore the closer the ROC curve to the point (0,1) in the figure the better the separability of the two distributions.

This attribute can be quantified by computing the AUROC (shown in gray). The greater the area, the greater the separability of the two distributions.

It is worth noting that this ROC analysis is based on a binary classifier with a single threshold. More complex analysis could involve a binary classifier with more than one threshold, or more complicated classifiers.

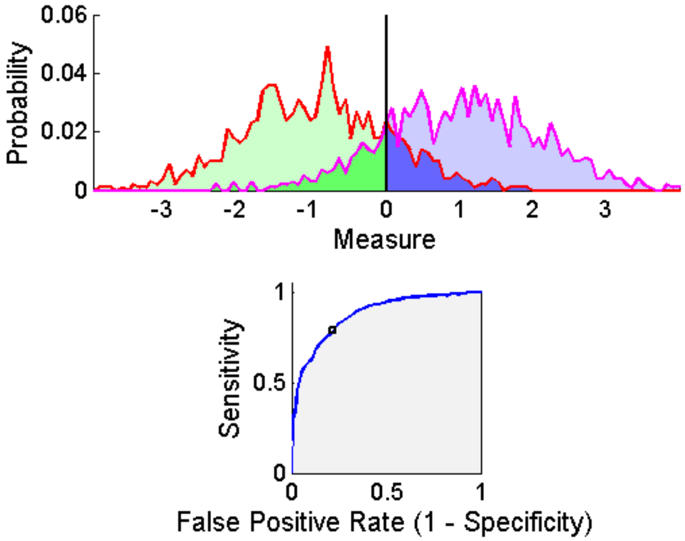

Figure 1. ROC analysis: Two distributions are discriminated using a variable threshold (top) in order to produce a ROC curve which plots sensitivity versus false positive rate (bottom). In the top plot, distributions A and B are red and magenta, respectively. Moreover, the proportions of TPs, FPs, TNs, and FNs correspond to the areas of the light blue, dark blue, light green, and green regions, respectively. The vertical black line represents a discrimination threshold. In the bottom plot, the ROC curve is blue and the area under the ROC curve is gray. In addition, the black square shows the point corresponding to where the threshold is positioned in the top plot.

2 Results

2.1 Example Time-Frequency Power Spectra

Here we present example spectra to illustrate the frequency characteristics of nitrous oxide inhalation. Example spectra during 60% peak gas induction for the subject presented in Figures 1, 2 and 3 of the manuscript are shown in Figures 2 and 3 for the common-reference and Laplacian-reference derivations, respectively. The spectra were calculated from intermediate steps in the global coherence analysis. For both figures gas induction starts at $t = 0$ minutes and reaches equilibrated gas levels after $t = 5$ minutes. The equilibrated gas period lasts until $t = 15$ minutes after which the nitrous oxide is removed for the rest of the recording. It can be seen that posterior alpha frequencies (around 11 Hz) are dominant in both the common-reference (Figure 2) and the Laplacian-reference derivations (Figure 3) during the initial period of inhalation where gas concentration is lower and also after the nitrous oxide is removed. Note there is a strong power rebound after gas removal. In addition, alpha power is present to a lesser degree in frontal electrodes for the common-reference derivation, largely due to volume conduction. Similar

spectral properties are observed for the 5 minute rest recording (not shown here). For the gas recording data in Figures 2 and 3, during the period of peak gas one can see intermittent weakening of the posterior alpha frequencies. Similar spectral properties during both the rest and gas recordings were observed with all subjects in the 60% peak gas group. Based on a previous power analysis of the data for the 20 and 40% peak gas groups [1], changes in alpha frequency are less noticable and increases in frontal delta and theta frequencies are more prevalent under nitrous oxide induction.

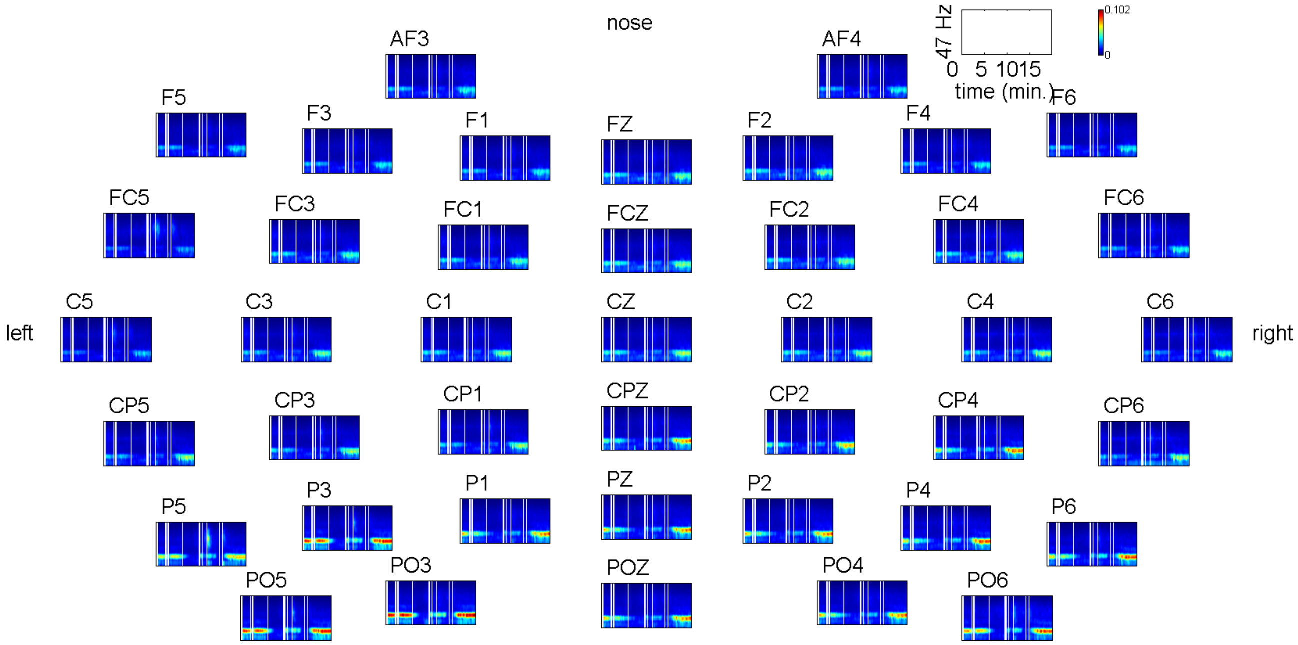

Figure 2. Power spectra time series during 60% peak gas induction for the same subject from Figures 1, 2 and 3 of the manuscript with the common-reference derivation. Channel labels are positioned above the corresponding spectra. The colorbar indicates power and for each spectra the y-axis corresponds to frequency which increases from 0 Hz at the bottom to 47 Hz at the top, and the x-axis corresponds to time in minutes increasing from $t = 0$ on the left to $t = 20$ minutes on the right. Vertical white spaces indicate times of removed artefact data.

2.2 Row Weight Snap Shots

Given that assessing the presence of globally coherent activity using a GC analysis depends not just on a high GC value but also the eigenvector/row weights corresponding to the largest eigenvalue, here we present example snap shots of the row weights for the same 60% peak gas subject as in Figures 1, 2 and 3 of the main paper. Figures 4 and 5 show the row weights obtained during rest and the last five minutes of the peak gas equilibration period for the full, frontal and parietal networks obtained with the common-reference and Laplacian reference derivations, respectively. For the rest data, GC at 11 Hz was generally high, therefore row weights are shown for the peak GC value at 11 Hz. For the gas data, GC at 11 Hz underwent intermittent decreases relative to magnitudes seen at rest, therefore row weights are shown for the times at which the maximal and minimal GC values occurred during the last five minutes of the peak gas equilibration period. Considering the full network with the common-reference derivation one can see that the coherent activity shifts more anterior when comparing rest with the gas case. This is true also

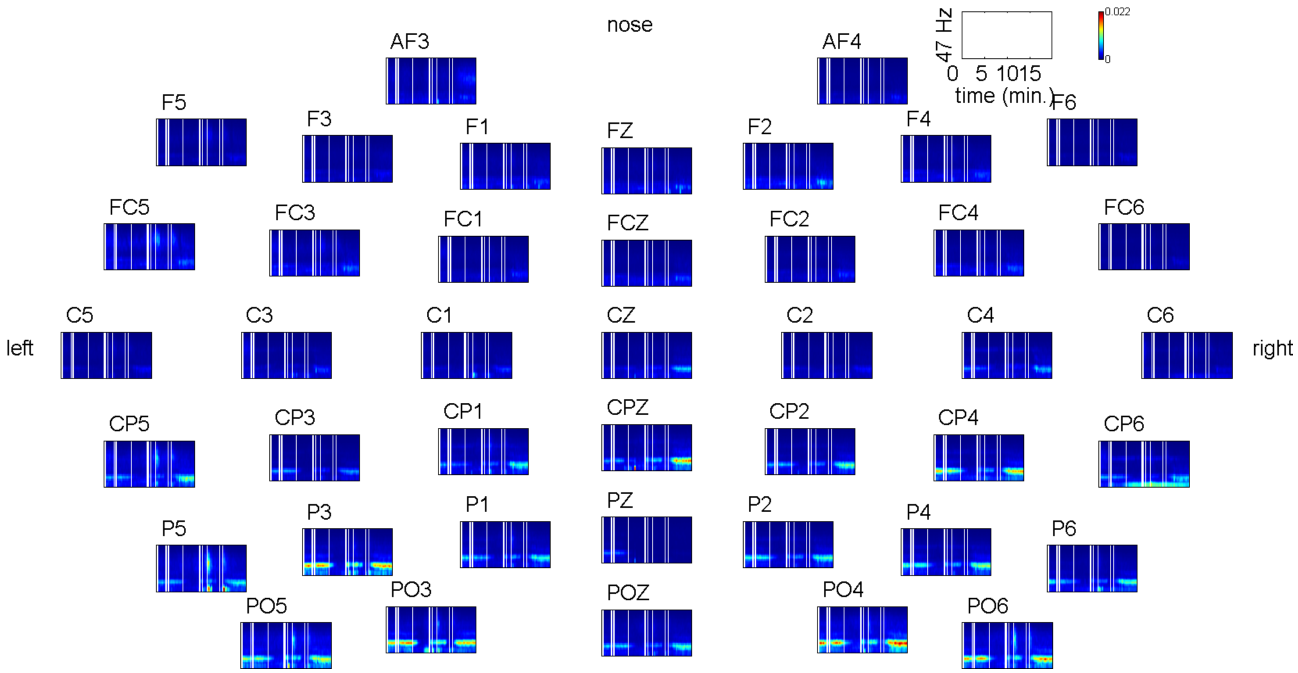

Figure 3. Power spectra time series during 60% peak gas induction for the same subject from Figures 1, 2 and 3 of the manuscript with the Laplacian-reference derivation. Figure details are similar to those of Figure 2.

for the full network with the Laplacian-reference derivation, although the shift does not appear to move as far forward and the spatial pattern is less diffuse. The row weight patterns for the frontal network do not appear to change greatly for this subject when comparing rest and gas for the common-reference and Laplacian-reference derivations. The row weight patterns for the parietal network appear to shift from a right bias to a left bias when going from rest to gas for the Laplacian-reference derivations. The row weight changes in the frontal and parietal networks were not considered in detail, but the anterior shift in coherent sites in the full brain network when going from rest to gas was consistent within all subjects within the 60% peak gas group and is considered in more detail in the following section.

2.3 Relationship Between Gas Concentration and Responsiveness

Here we illustrate that high N_2O gas concentrations were better correlated to lower aCPT accuracy and longer reaction times than were the lower gas concentrations. This is consistent with a reduction in consciousness at high N_2O gas concentrations. Figure 6A and B show the dependence on gas concentration for aCPT accuracy and reaction time, respectively. In both subfigures, as gas concentration increases, accuracy becomes lower (i.e. there is a greater proportion of 'incorrect' responses) and reaction times also increase.

2.4 Combined 20%, 40% and 60% Peak Gas Group Results

2.4.1 Combined group data dependence on gas concentration

Figures 7, 8 and 9 demonstrate that there are minimal network changes for the combined group for gas concentrations below 40%, and for higher gas concentrations the network-based AUROC patterns observed are the same as those for the 60% peak gas group in Figure 7 in the manuscript. Figure 7 shows box-whisker plots for GE-based functional connectivity (E_W) defined relative to the median during rest for the full brain (top row), frontal (middle row) and parietal (bottom row) networks obtained either with common-reference (left column) or Laplacian re-referencing (right column). It can be seen that weaker changes occur, if any, for gas concentrations below 40% when compared to the 60% peak gas group alone in Figure 5 of the manuscript.

Figure 8 shows box-whisker plots for GC-based functional connectivity (C_G) at 11 Hz defined relative to the median during rest for the full brain (top row), frontal (middle row) and parietal (bottom row) networks obtained either with common-reference (left column) or Laplacian re-referencing (right column). Again weaker changes occur, if any, for gas concentrations below 40% when compared to the 60% peak gas group alone (see Figure 6 of the manuscript).

Figure 9 summarises the results of the ROC analysis for all GE and GC measures as a function of gas concentration for the combined group, where AUROC score bars point up or down if the difference in the corresponding median measure value relative to rest reflects an increase or decrease, respectively. It can be seen that the AUROC pattern changes for high gas concentrations are the same as those seen in Figure 7 of the manuscript, and there is little network change occurring for gas concentrations below 40% based on the measures considered.

2.4.2 Combined group data dependence on responsiveness

The dependence of the measures on auditory task performance accuracy for the combined 20, 40 and 60% peak gas groups are presented in Figure 10 for GE (E_W) and in Figure 11 for GC (C_G). In both figures it can be seen that although there are slight differences in the measures between 'rest' and 'incorrect', there is still strong overlap. The associated ROC analysis results are shown in Figure 12. Only weakly significant ROC differences were found for the measures dependent on auditory task performance accuracy. The most significant changes were increases in full brain GE measures E_w and e_{top} for the common-reference with decreases in auditory task performance.

The dependence of the measures on auditory task performance reaction time for the combined 20, 40 and 60% peak gas groups are presented in Figure 13 for GE (E_W) and in Figure 14 for GC (C_G). In both figures, the reaction time bins correspond to data collected from the gas recording. Moreover, in both figures it can be seen that for longer reaction times the measures are more separable from 'rest' than for auditory task performance accuracy. The associated ROC analysis results are shown in Figure 15. Compared to auditory task accuracy, more significant AUROC differences were found for the measures dependent on long reaction times. The most significant changes were increases in full brain E_w for the common-reference and decreases in full brain and parietal E_w for the Laplacian-reference, with increases in auditory task reaction time.

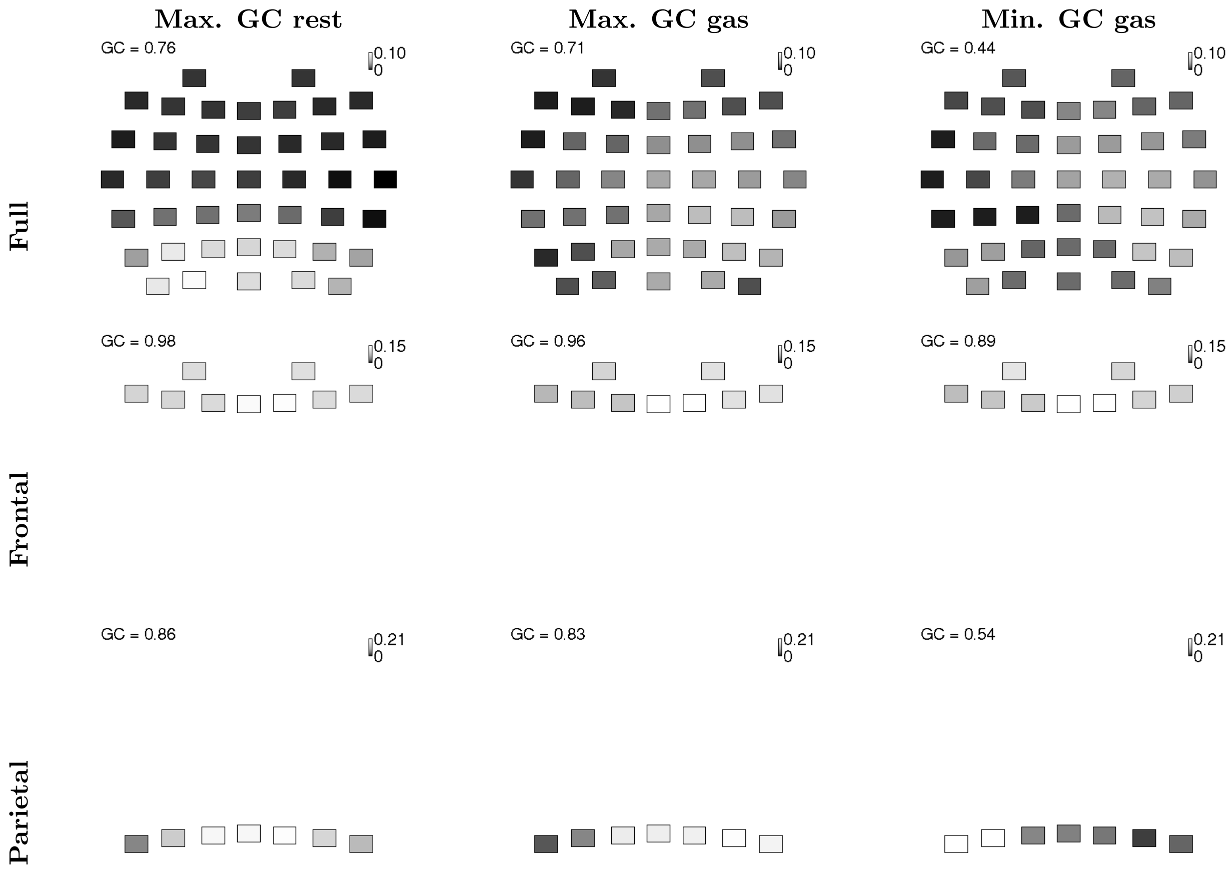

Figure 4. Snapshots of the row weights at 11 Hz for the full brain (top row), frontal (middle row) and parietal (bottom row) networks for the common-reference derivation for the same subject from Figures 1, 2 and 3 of the manuscript and the 60% peak gas group. Snapshots are shown at times of maximal GC value during rest (left column), and the maximal (middle column) and minimal (right column) GC values during the last 5 minutes of the peak gas equilibration period of the gas recording. The associated GC value is positioned at the top left of each row weight map, and the colormap is given at the top right. The top corresponds to the front of the head, and the left and right correspond to the left and right of the head, respectively.

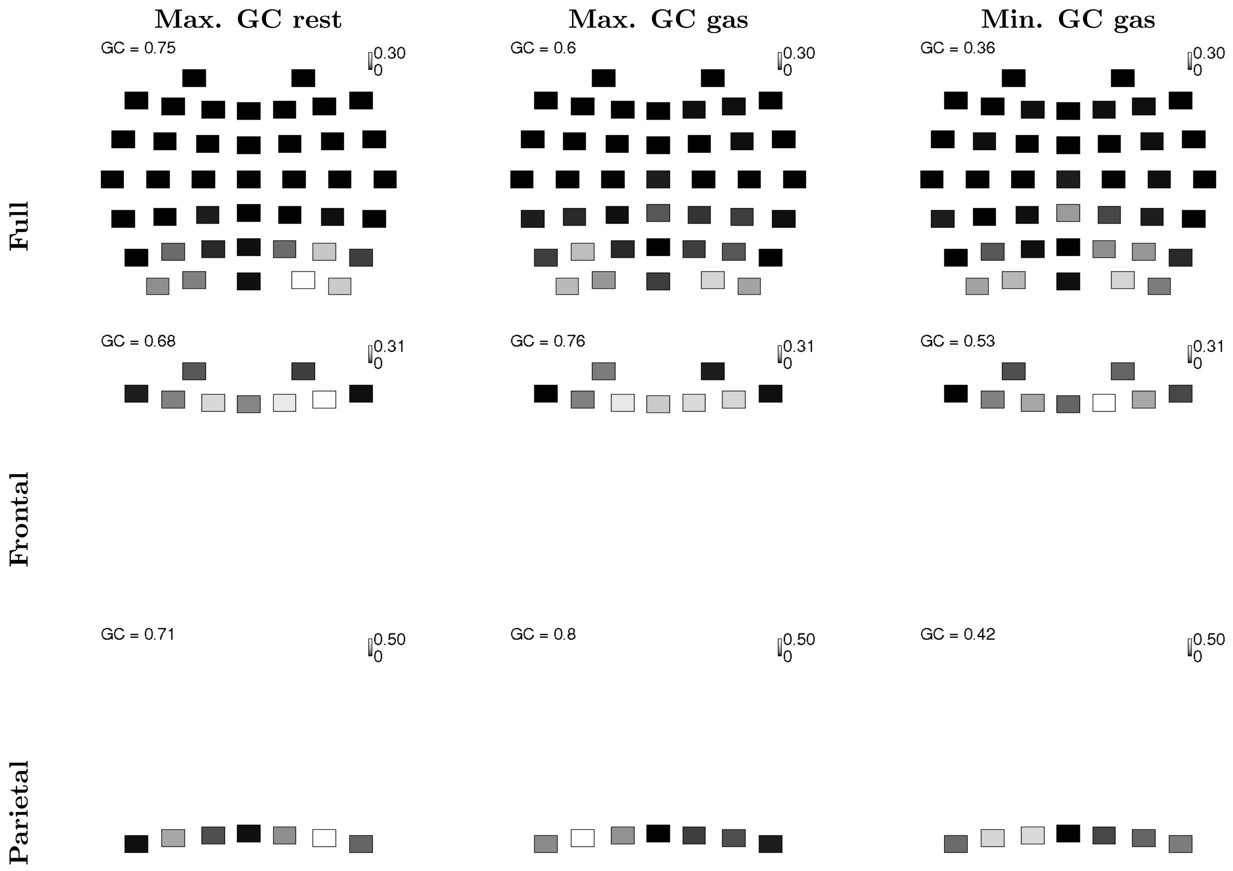

Figure 5. Snapshots of the row weights at 11 Hz for the full brain (top row), frontal (middle row) and parietal (bottom row) networks for the Laplacian-reference derivation for the same subject from Figures 1, 2 and 3 of the manuscript and the 60% peak gas group. Snapshots are shown at times of maximal GC value during rest (left column), and the maximal (middle column) and minimal (right column) GC values during the last 5 minutes of the peak gas equilibration period of the gas recording. The associated GC value is positioned at the top left of each row weight map, and the colormap is given at the top right. The top corresponds to the front of the head, and the left and right correspond to the left and right of the head, respectively.

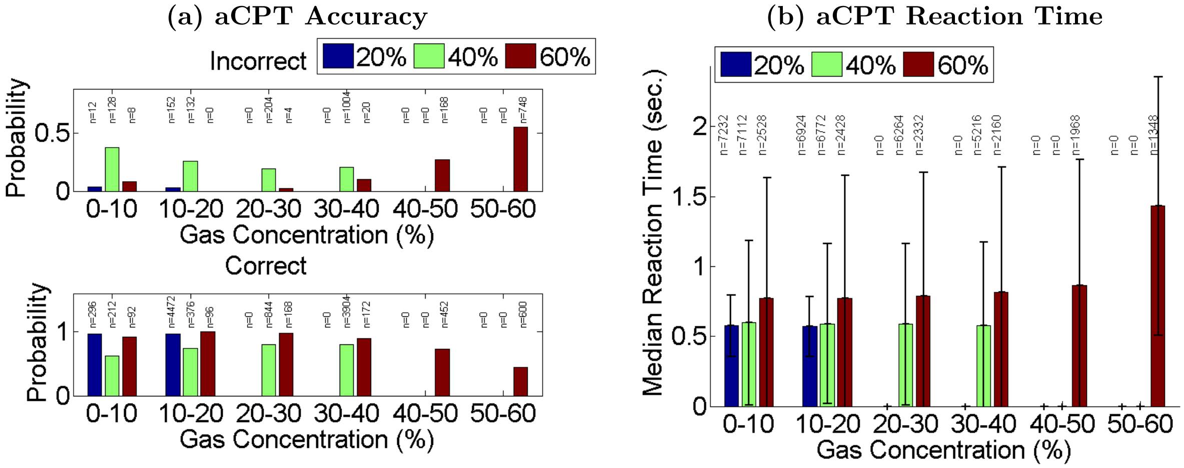

Figure 6. Relationship Between N_2O Gas Concentration and Responsiveness. (A) Dependence of aCPT accuracy on reaction time. (B) Dependence of median aCPT reaction time on gas concentration. In (A) the top and bottom y-axes represent the probability of 'incorrect' and 'correct', respectively, for a given gas concentration bin. (B) error bars indicate ± one standard deviation. The legend indicates different peak gas concentration groups.

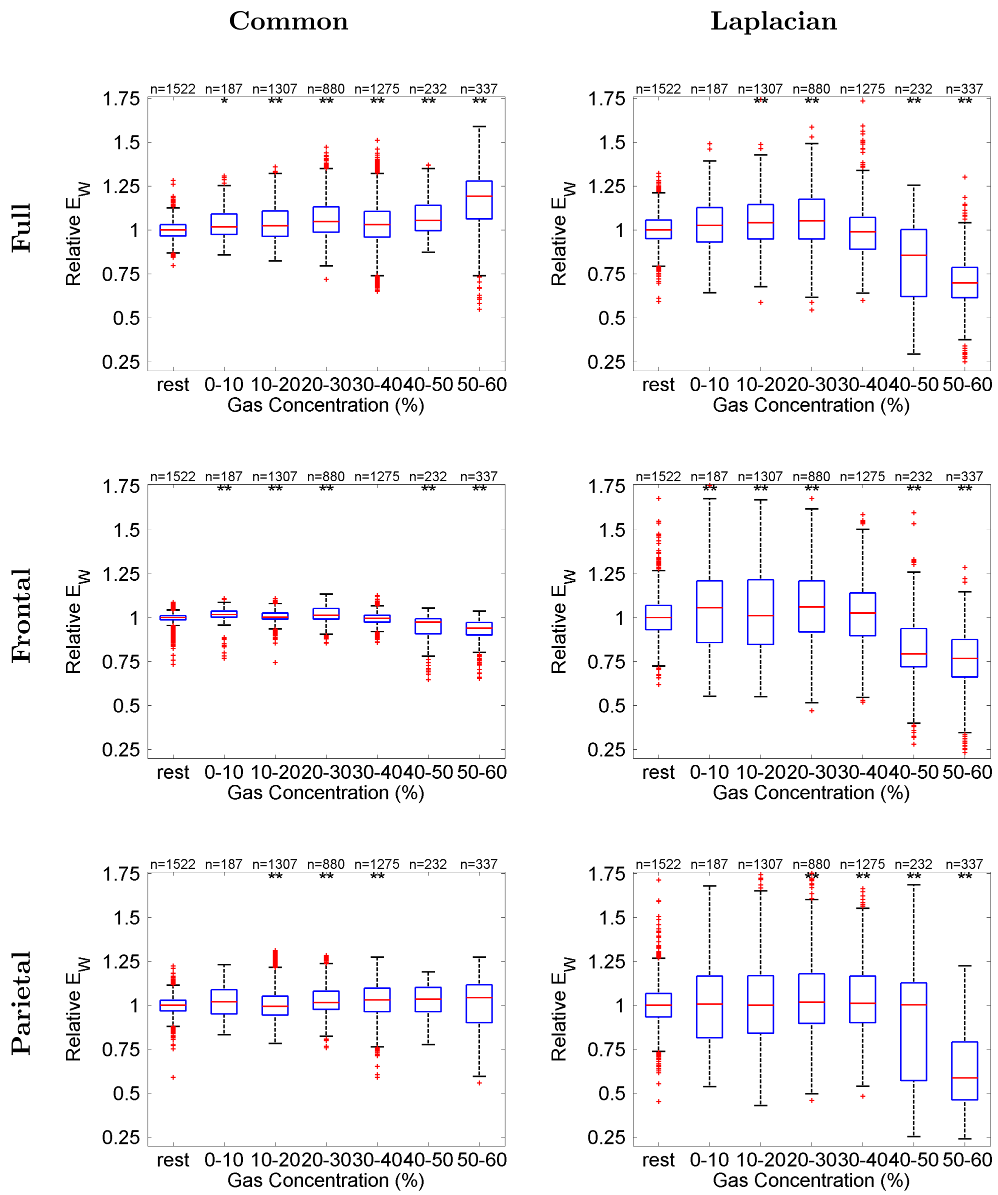

Figure 7. Dependence, for the combined data, of GE-based functional connectivity (E_W) defined relative to the median during rest on N_2O gas concentration for full brain (top row), frontal (middle row) and parietal (bottom row) networks, obtained either with common-reference (left column) or Laplacian re-referencing (right column). Box-whisker and multi-comparison test significance marker ($*$,$**$) details are the same as for Figure 5 in the manuscript.

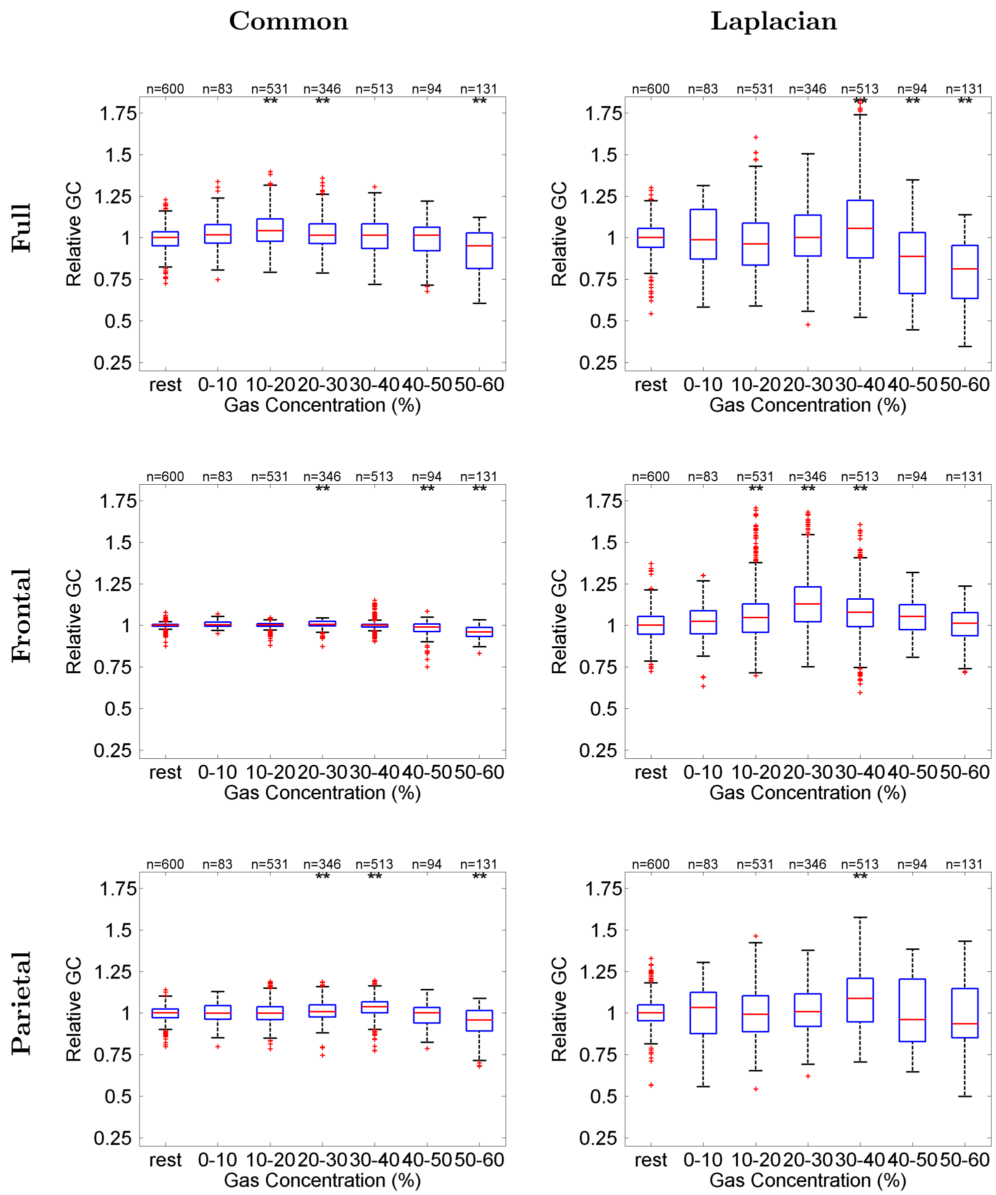

Figure 8. Dependence, for the combined data, of GC-based functional connectivity (C_G) at 11 Hz defined relative to the median during rest on N_2O gas concentration for full brain (top row), frontal (middle row) and parietal (bottom row) networks, obtained either with common-reference (left column) or Laplacian re-referencing (right column). Box-whisker and multi-comparison test significance marker ($*$,$**$) details are the same as for Figure 5.

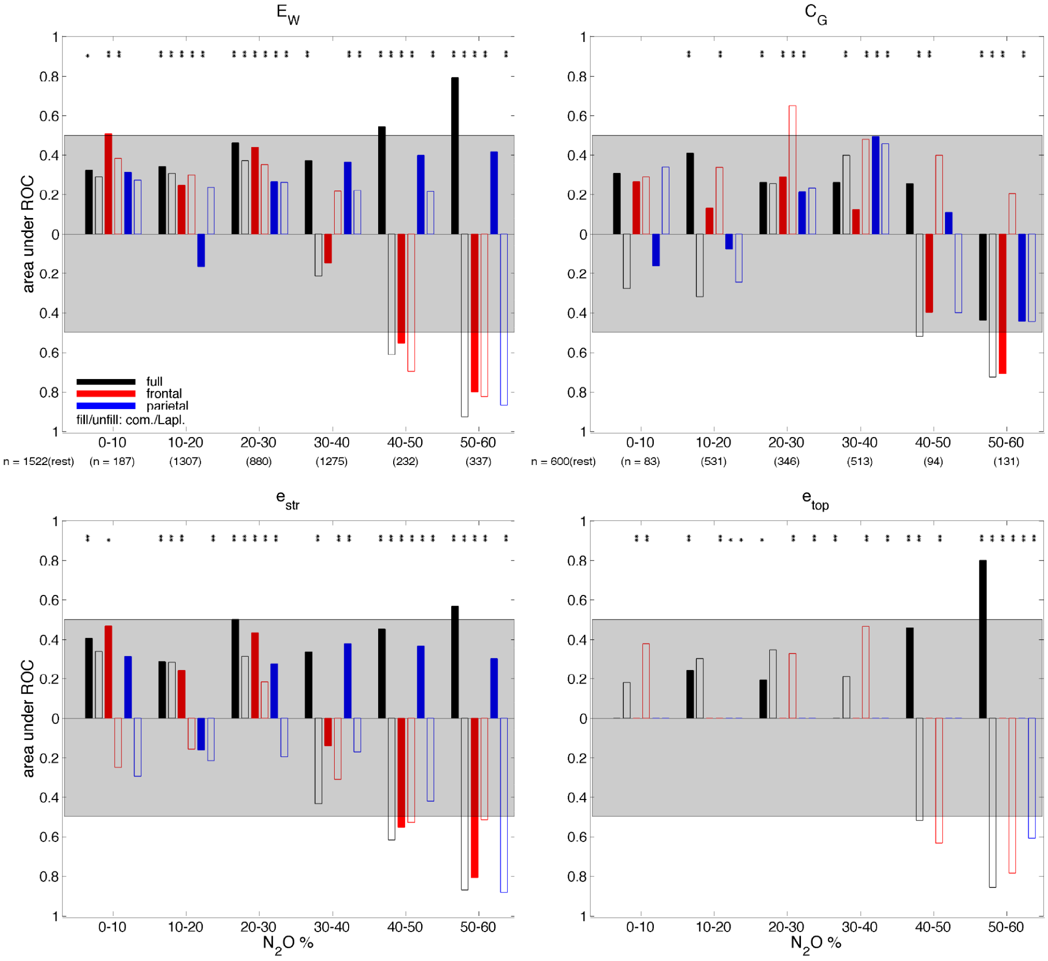

Figure 9. Area under the receiver operating characteristic curve (AUROC) as a function of inspired N_2O concentration for the combined data for (top left) weighted global efficiency, E_W, (top right) global coherence, C_G, at 11 Hz, (bottom left) the contribution of connection strength to global efficiency, e_{str}, and (bottom right) the contribution of connection topology to global efficiency, e_{top}. One-way ANOVAs were significant across rest and the six gas concentration bins for each of the measures ($p < 10^{-12}$). The difference in the median measure value relative to rest is indicated by the the direction of the respective bar (up, increase; down, decrease). Multi-comparison test significance marker ($*$,$\overset{*}{*}$) details are the same as for Figure 5. The remaining features are the same as described in Figure 7.

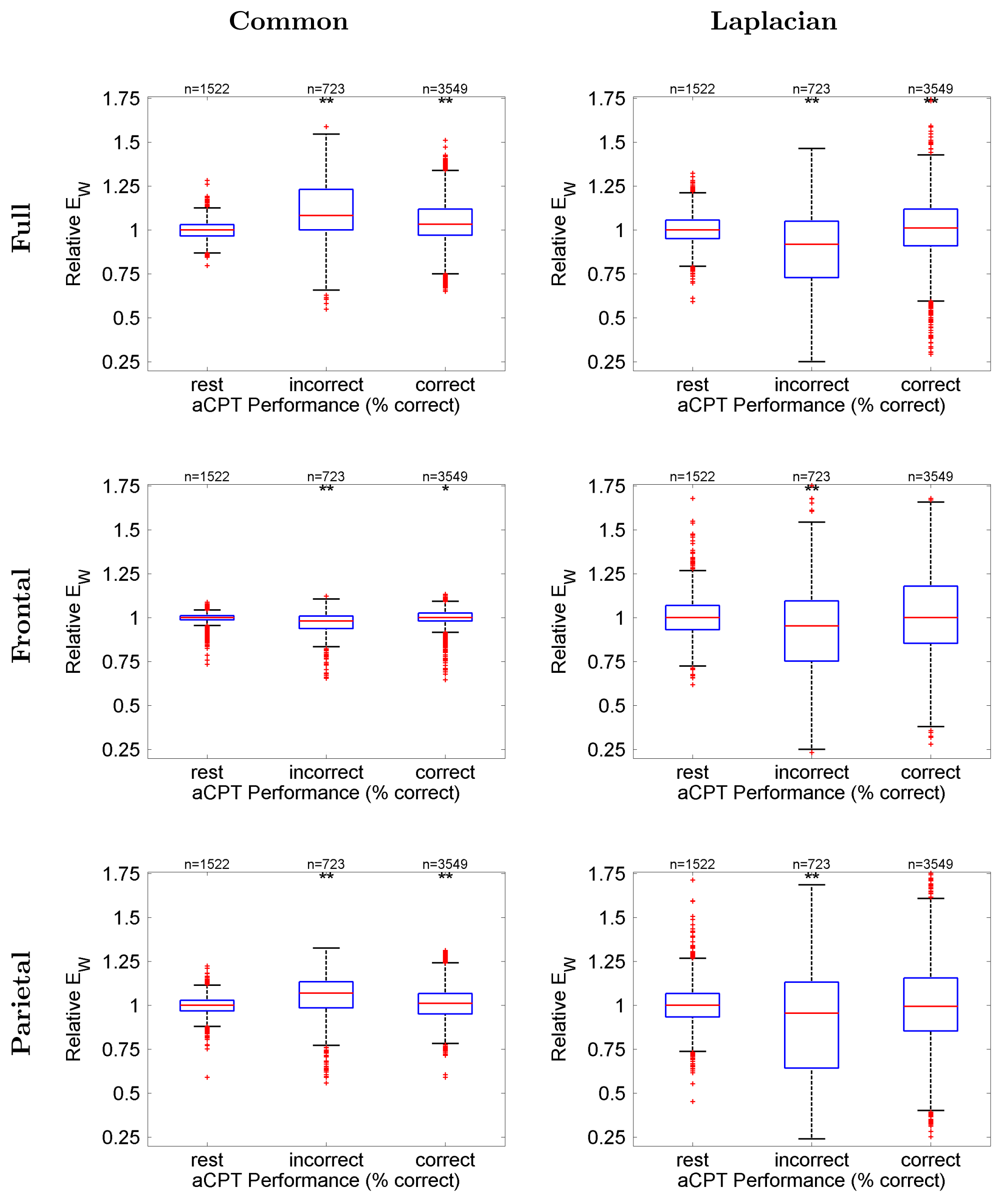

Figure 10. Dependence, for the combined data, of GE-based functional connectivity (E_W) defined relative to the median during rest on aCPT accuracy for full brain (top row), frontal (middle row) and parietal (bottom row) networks, obtained either with common-reference (left column) or Laplacian re-referencing (right column). Box-whisker and multi-comparison test significance marker ($*$,$**$) details are the same as for Figure 5.

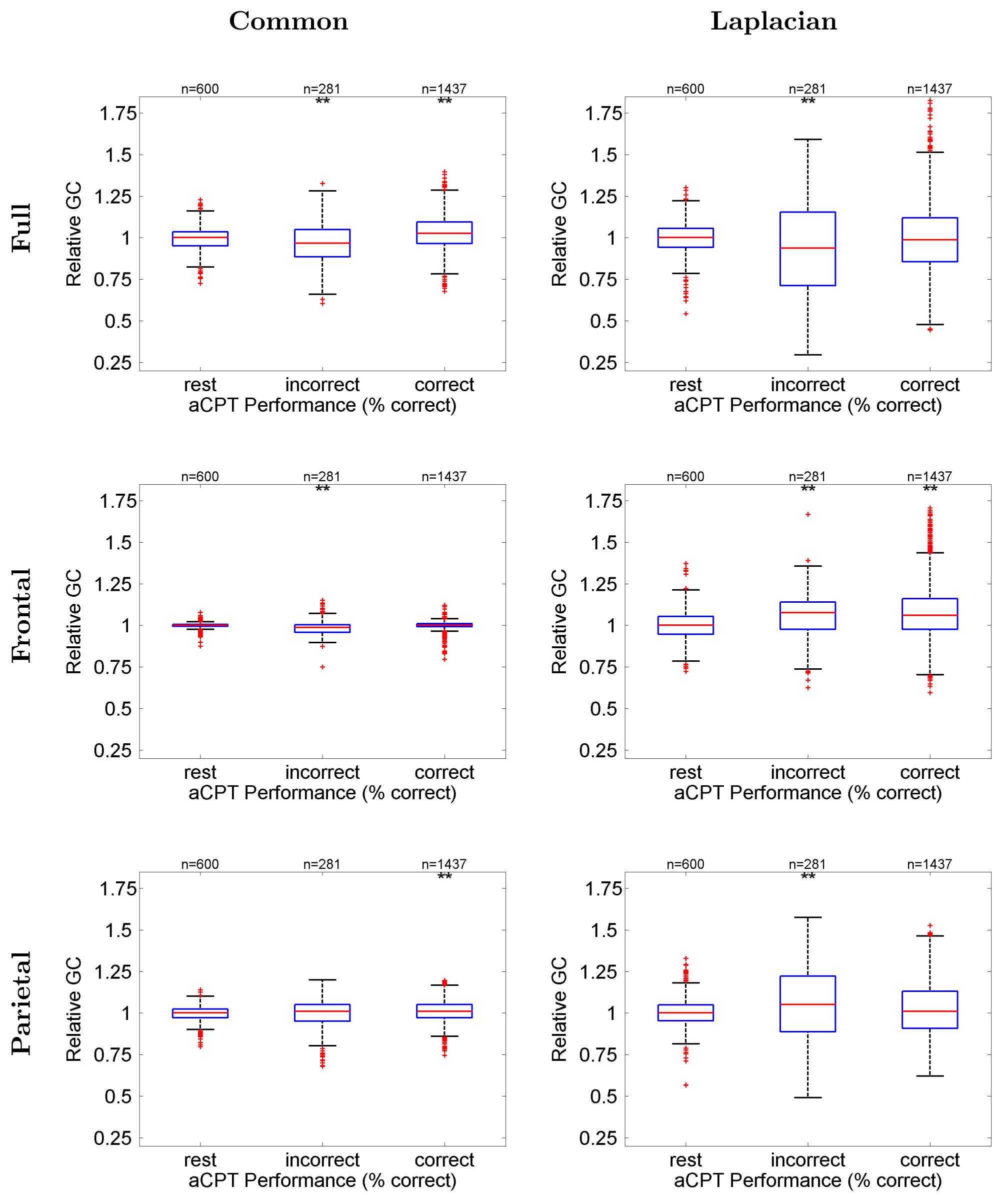

Figure 11. Dependence, for the combined data, of GC-based functional connectivity (C_G) at 11 Hz defined relative to the median during rest on aCPT accuracy for full brain (top row), frontal (middle row) and parietal (bottom row) networks, obtained either with common-reference (left column) or Laplacian re-referencing (right column). Box-whisker and multi-comparison test significance marker ($*$,$**$) details are the same as for Figure 5.

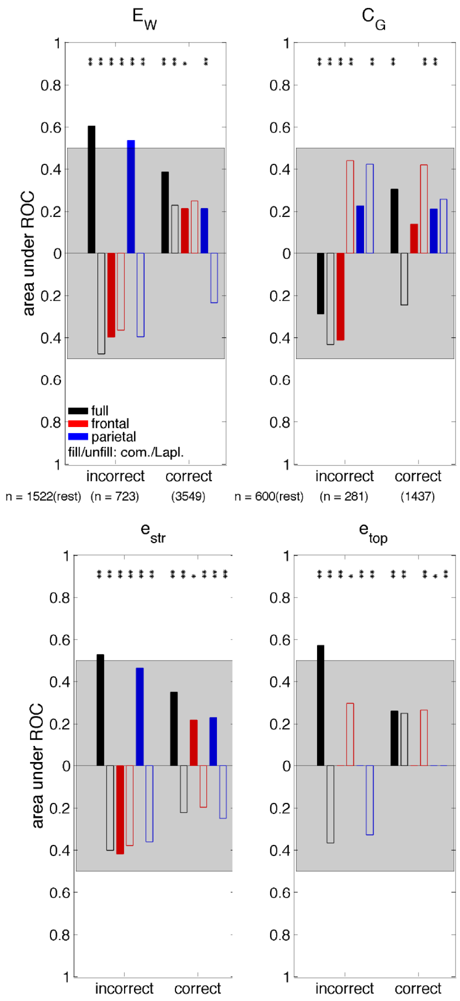

Figure 12. AUROC as a function of aCPT accuracy for the combined data for (top left) weighted global efficiency, E_W, (top right) global coherence, C_G, at 11 Hz, (bottom left) the contribution of connection strength to global efficiency, e_{str}, and (bottom right) the contribution of connection topology to global efficiency, e_{top}. One-way ANOVAs were significant across rest and the two accuracy bins for each of the measures ($p < 0.0008$ for all cases). The difference in the median measure value relative to rest is indicated by the the direction of the respective bar (up, increase; down, decrease). Multi-comparison test significance marker ($*$,$\overset{*}{*}$) details are the same as for Figure 5. The remaining features are the same as described in Figure 7.

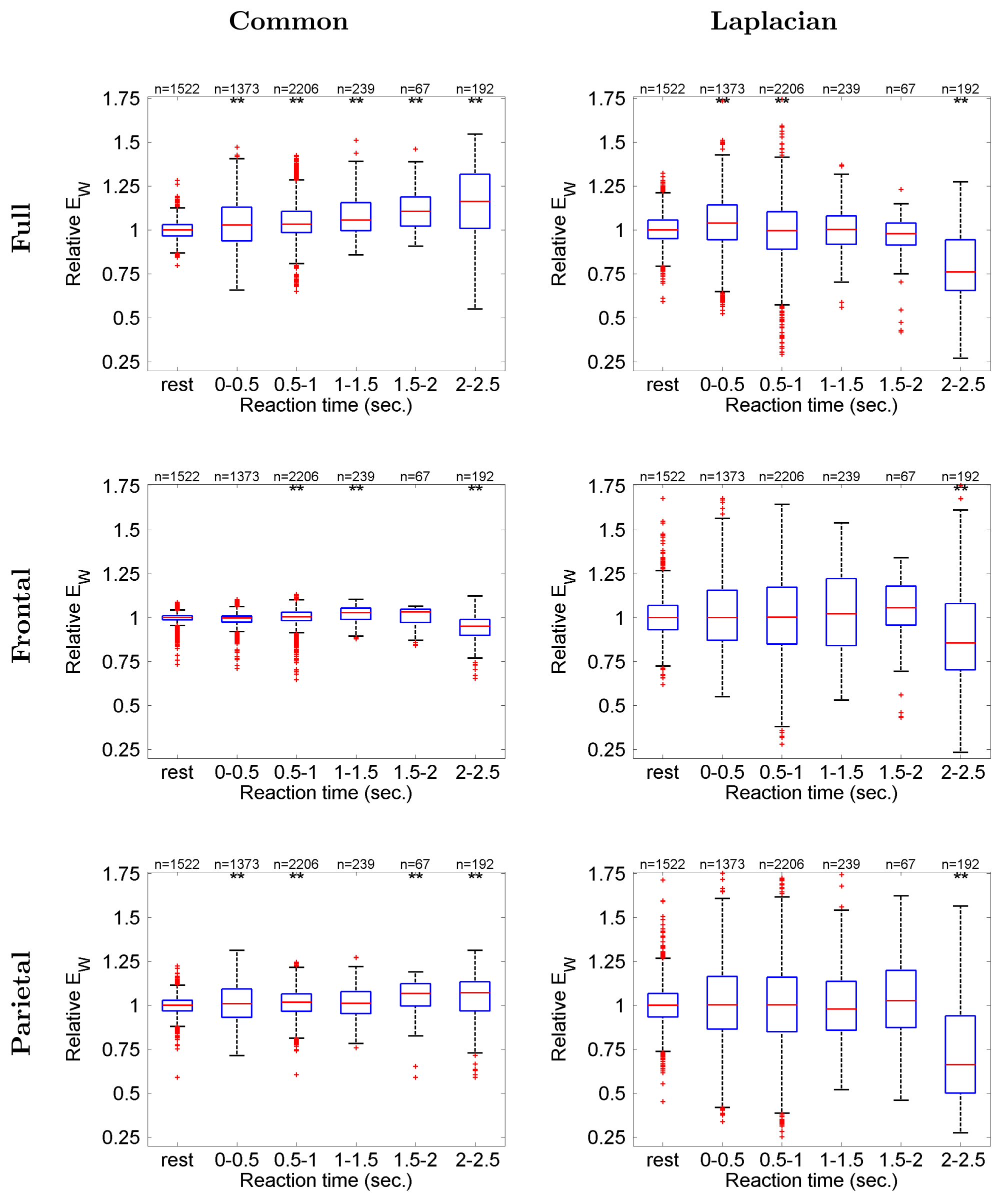

Figure 13. Dependence, for the combined data, of GE-based functional connectivity (E_W) defined relative to the median during rest on aCPT reaction time for full brain (top row), frontal (middle row) and parietal (bottom row) networks, obtained either with common-reference (left column) or Laplacian re-referencing (right column). Box-whisker and multi-comparison test significance marker ($*$,$**$) details are the same as for Figure 5.

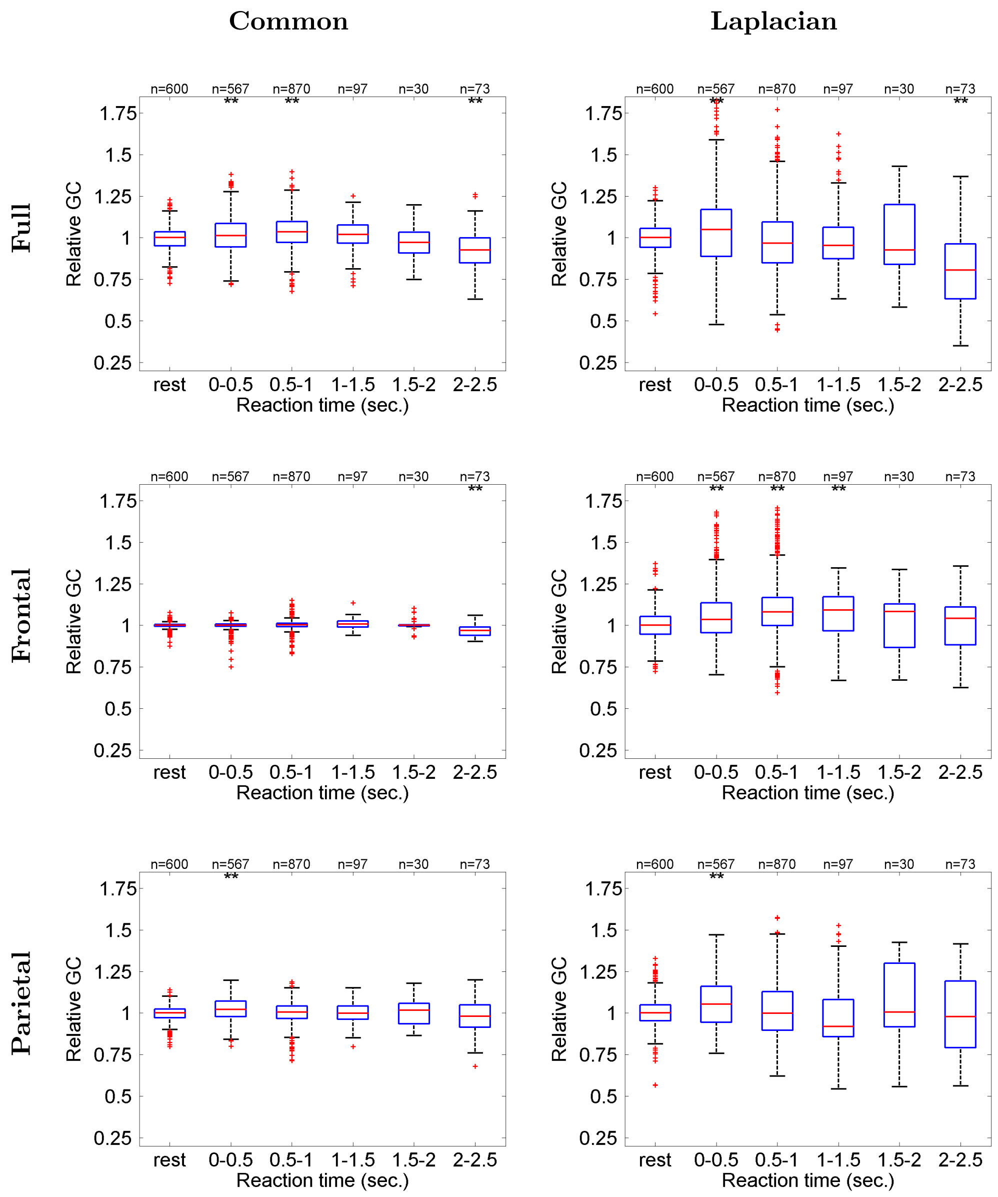

Figure 14. Dependence, for the combined data, of GC-based functional connectivity (C_G) at 11 Hz defined relative to the median during rest on aCPT reaction time for full brain (top row), frontal (middle row) and parietal (bottom row) networks, obtained either with common-reference (left column) or Laplacian re-referencing (right column). Box-whisker and multi-comparison test significance marker ($*$,$**$) details are the same as for Figure 5.

2.5 60% Peak Gas Group Box Whisker Plots for Behavioural Data

The multi-scale multi-network dependence of GE-based functional connectivity and GC-based functional connectivity at 11 Hz on aCPT accuracy for the 60% peak gas group is illustrated in Figures 16 and 17, respectively. Unresponsiveness corresponds to the 'incorrect' case since the auditory task was easy and incorrect responses were rarely obtained in the rest state. Similar changes that were observed for high gas concentrations were also observed for the 'incorrect' case.

The multi-scale multi-network dependence of GE-based functional connectivity and GC-based functional connectivity at 11 Hz on aCPT reaction times for the 60% peak gas group is illustrated in Figures 18 and 19, respectively. Again, similar changes that were observed for high gas concentrations were also observed for longer reaction times.

2.6 Additional Global Coherence and Power Analysis

An alternative way of viewing the global coherence data is to look at the cumulative row weight (CRW) pattern for a specific frequency (see definition of row weights in the methods) which provides a visual encoding of the principle electrodes that contribute to globally coherent activity and whether or not coherent activity is more posterior or anterior. For the same 60% peak gas subject as in Figures 1-4 of the main paper, Figure 20 shows CRW (top row) and cumulative power (CP - bottom row) plots for the 11 Hz frequency band obtained either with Common-reference (left column) or Laplacian re-referencing (right column). In the CRW plots for a given segment of data, the cumulative sum of the row weights is plotted vertically in increments starting the sum from the largest row weight down to the smallest row weight. Electrodes, which are assigned row weights, are mapped to specific colors as shown in the color map. Red colors indicate frontal electrodes whereas blue colors indicate posterior electrodes. For each added row weight, a cross the color of the corresponding electrode is plotted at the value of the associated cumulative sum. A similar method is applied for the CP plots, however, the average signal power associated with each electrode replaces the row weight values. CP is not based on the cross-spectrum and therefore acts as a control against cross-spectral changes. In each sub-figure the measured end-tidal N_2O gas concentrations are plotted in black and the auditory task performance is plotted in magenta. It can be noted that close to rest periods coherent activity and power are predominantly posterior as indicated by the blue crosses, whereas during the equilibrated gas period there are intermittent shifts towards the front as indicated by the darker green and red colors. With the common-reference CRW and CP both show a high mixture of frontal and posterior activity during transition to peak gas. With Laplacian re-referencing CRW and CP both show sharper tuning and a greater posterior dominance.

To quantify changes in anteriorisation/posteriorisation the measures dRWS and dPS were considered (see Section 1.1). For the 60% peak gas group data, Figure 21 illustrates the dependence of dRWS and dPS on N_2O gas concentration through box whisker plots for dRWS (top row) and dPS (bottom row) defined relative to the median value of the rest data, obtained either with Common-reference (left column) or Laplacian re-referencing (right column). In both cases it can be seen that dRWS and dPS generally increase as gas concentration increases with greater change seen with the Laplacian for dPS.

Table S1 summarises ROC analysis results for dRWS and dPS as a function of gas concentration for the 60% peak gas group data. The dRWS and dPS both show more significant changes with the Laplacian (dPS is more significant) pointing to changes occurring on smaller spatial scales in superficial cortical networks. These dRWS and dPS changes both involve increases with increasing gas concentrations, indicating a slight anteriorisation with higher gas concentrations (see Figure 21).

For the 60% peak gas group data, Tables S2 and S3 summarise ROC analysis results for dRWS and dPS as a function of auditory task performance accuracy and reaction time, respectively. As was the case for changes as a function of gas concentration, the dRWS and dPS both show more significant changes with the Laplacian. Of note is that dPS for the Laplacian-reference derivation showed the greatest significance for the longest reaction time for the combined dataset compared to any of the measures

considered in this study (AUROC=0.99).

Table 1. AUROC statistics for dependence on N_2O gas concentration for measures of anteriorisation/posteriorisation

Measure	Ref.	AUROC for different gas concs. vs rest					
		0-10%	10-20%	20-30%	30-40%	40-50%	50-60%
dRWS	C	**0.55** ↑	n/a	0.35	0.37	**0.67** ↑	**0.66** ↑
dRWS	L	0.05	n/a	0.25	0.24	**0.57** ↑	**0.69** ↑
dPS	C	0.53	n/a	0.19	0.49	**0.64** ↑	**0.68** ↑
dPS	L	0.29	n/a	**0.55** ↓	**0.51** ↓	**0.78** ↑	**0.84** ↑

C: Common reference; L: Laplacian reference. One-way ANOVAs were significant across rest and the six gas concentration bins for each measure ($p < 10^{-12}$). Bold highlights AUROC $\geq$ 0.5. Arrows to the right of significant bold AUROC scores indicate the difference in the median measure value relative to rest (↑, increase; ↓, decrease).

Table 2. AUROC statistics for dependence on auditory task performance accuracy for measures of anteriorisation/posteriorisation.

Measure	Ref.	AUROC of performance vs rest	
		incorrect	correct
dRWS	C	**0.60** ↑	**0.55** ↑
dRWS	L	**0.75** ↑	0.44
dPS	C	**0.69** ↑	**0.51** ↑
dPS	L	**0.95** ↑	**0.55** ↑

C: Common reference; L: Laplacian reference. One-way ANOVAs were significant across rest and the two task accuracy bins for each measure ($p < 10^{-12}$). Bold highlights AUROC $\geq$ 0.5. Arrows to the right of significant bold AUROC scores indicate the difference in the median measure value relative to rest (↑, increase; ↓, decrease).

2.7 Analysis involving subsampling down to 10:20 Montage

It is worthwhile to check if similar results are obtained when subsampling our electrodes down to the more commonly used 10:20 montage. When this is done for the 60% peak gas group, quite similar results are obtained. For example, Figures 22 and 23 illustrate for the 10:20 montage the dependence of GE and GC as a function of N_2O gas concentration, respectively. The changes observed are similar to those seen in Figures 4 and 5 of the main paper where the extended 10:20 montage has been used. It is important to note that in Figures 22 and 23, for the column corresponding to Common-reference derivations, all 21 channels of the 10:20 system were analysed, whereas for the column corresponding to Laplacian re-referenced derivations, only 13 channels were analysed as a result of the Laplacian. Despite this small

Table 3. AUROC statistics for dependence on auditory task reaction times for measures of anteriorisation/posteriorisation.

Measure	Ref.	AUROC of reaction time vs rest				
		0-50ms	50-100ms	100-150ms	150-200ms	200-250ms
dRWS	C	0.49	**0.63** ↑	**0.62** ↑	n/a	**0.67** ↑
dRWS	L	0.33	**0.51** ↑	**0.71** ↑	n/a	**0.81** ↑
dPS	C	0.37	**0.65** ↑	**0.76** ↑	n/a	**0.79** ↑
dPS	L	**0.53** ↓	**0.67** ↑	**0.75** ↑	n/a	**0.99** ↑

C: Common reference; L: Laplacian reference. One-way ANOVAs were significant across rest and the two task accuracy bins for each measure ($p < 10^{-11}$). Bold highlights AUROC ≥ 0.5. Arrows to the right of significant bold AUROC scores indicate the difference in the median measure value relative to rest (↑, increase; ↓, decrease).

number of channels, results are quite similar to the case with greater electrode density shown in the main paper.

References

[1] Foster BL, Liley DTJ (2011) Nitrous Oxide Paradoxically Modulates Slow Electroencephalogram Oscillations: Implications for Anesthesia Monitoring. *Anesth Analg* 113(4):758-765.

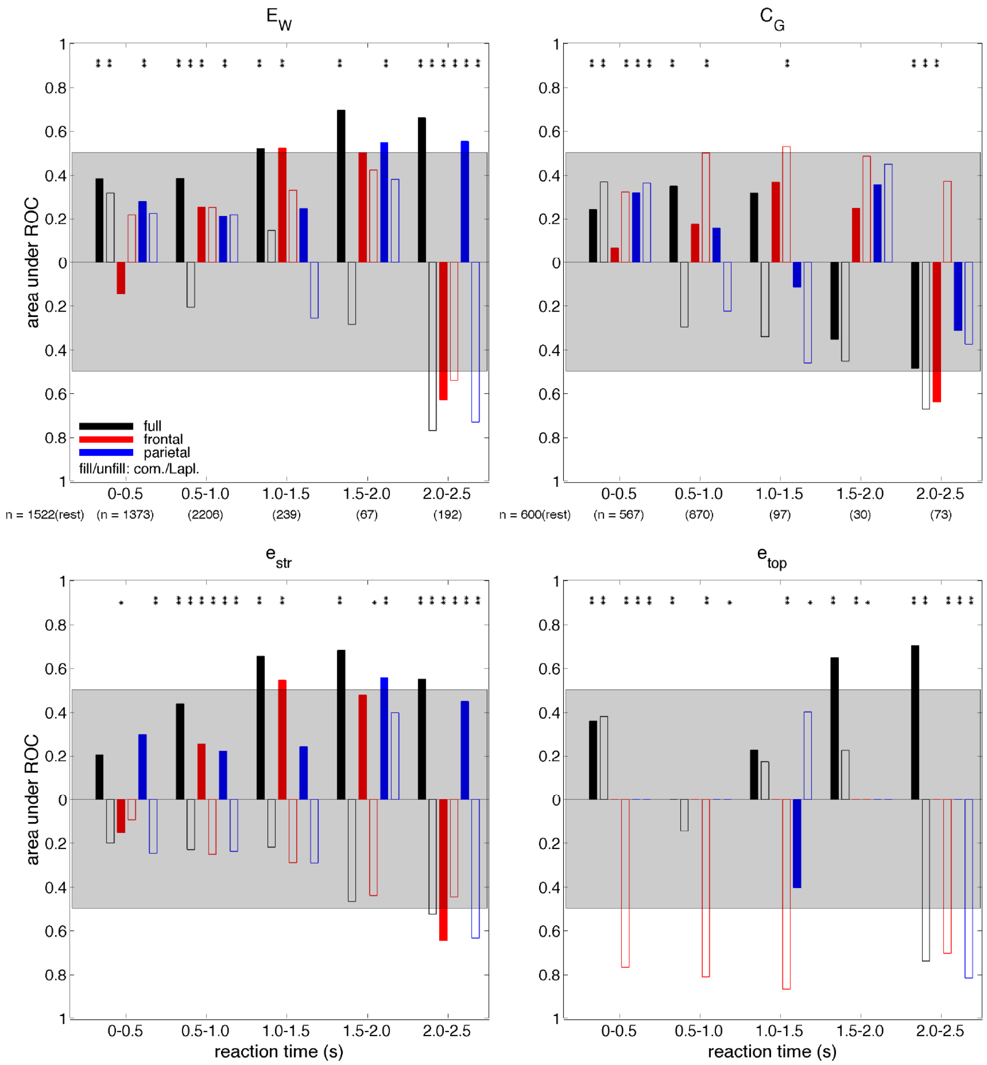

Figure 15. AUROC as a function of aCPT reaction time for the combined data for (top left) weighted global efficiency, E_W, (top right) global coherence, C_G, at 11 Hz, (bottom left) the contribution of connection strength to global efficiency, e_{str}, and (bottom right) the contribution of connection topology to global efficiency, e_{top}. One-way ANOVAs were significant across rest and the five reaction time bins for each of the measures ($p < 10^{-4}$ for all cases). The difference in the median measure value relative to rest is indicated by the the direction of the respective bar (up, increase; down, decrease). Multi-comparison test significance marker ($*$,$\overset{*}{*}$) details are the same as for Figure 5. The remaining features are the same as described in Figure 7.

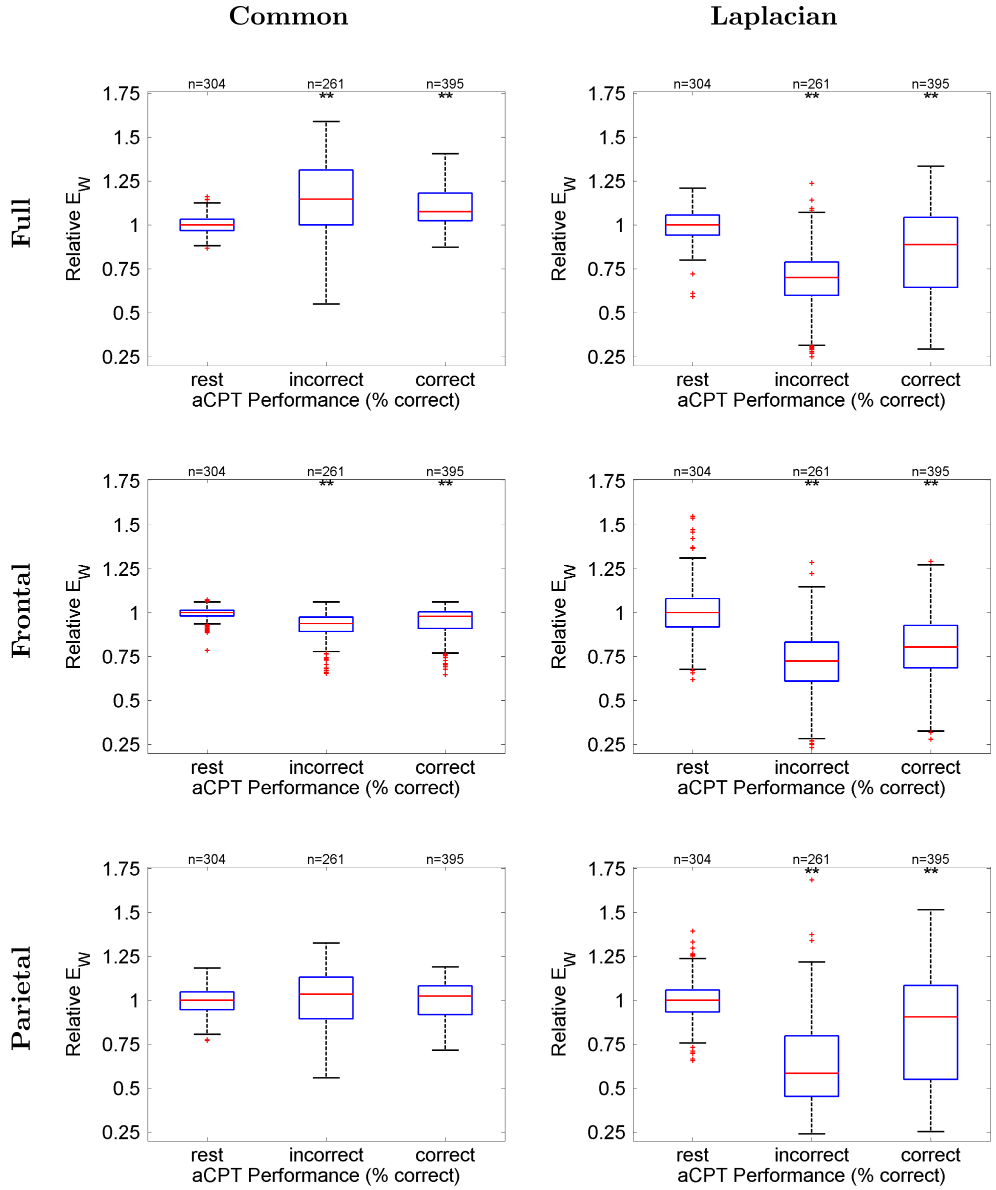

Figure 16. Dependence, for the 60% peak gas group data, of GE-based functional connectivity (E_W) defined relative to the median during rest on aCPT accuracy for full brain (top row), frontal (middle row) and parietal (bottom row) networks, obtained either with common-reference (left column) or Laplacian re-referencing (right column). Box-whisker and multi-comparison test significance marker (∗,∗∗) details are the same as for Figure 5 in the manuscript.

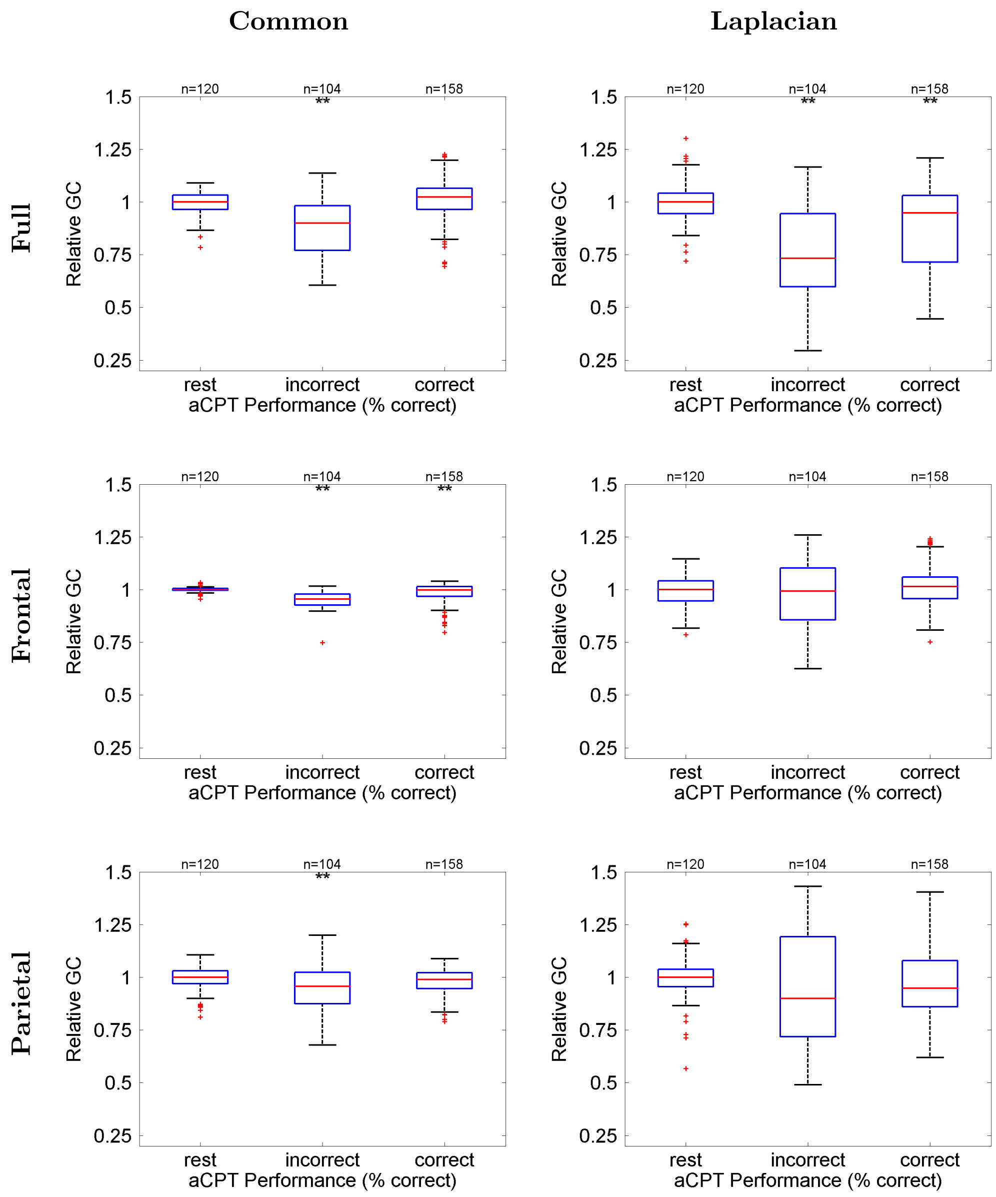

Figure 17. Dependence, for the 60% peak gas group data, of GC-based functional connectivity (C_G) at 11 Hz defined relative to the median during rest on aCPT accuracy for full brain (top row), frontal (middle row) and parietal (bottom row) networks, obtained either with common-reference (left column) or Laplacian re-referencing (right column). Box-whisker and multi-comparison test significance marker ($*$,$**$) details are the same as for Figure 5 in the manuscript.

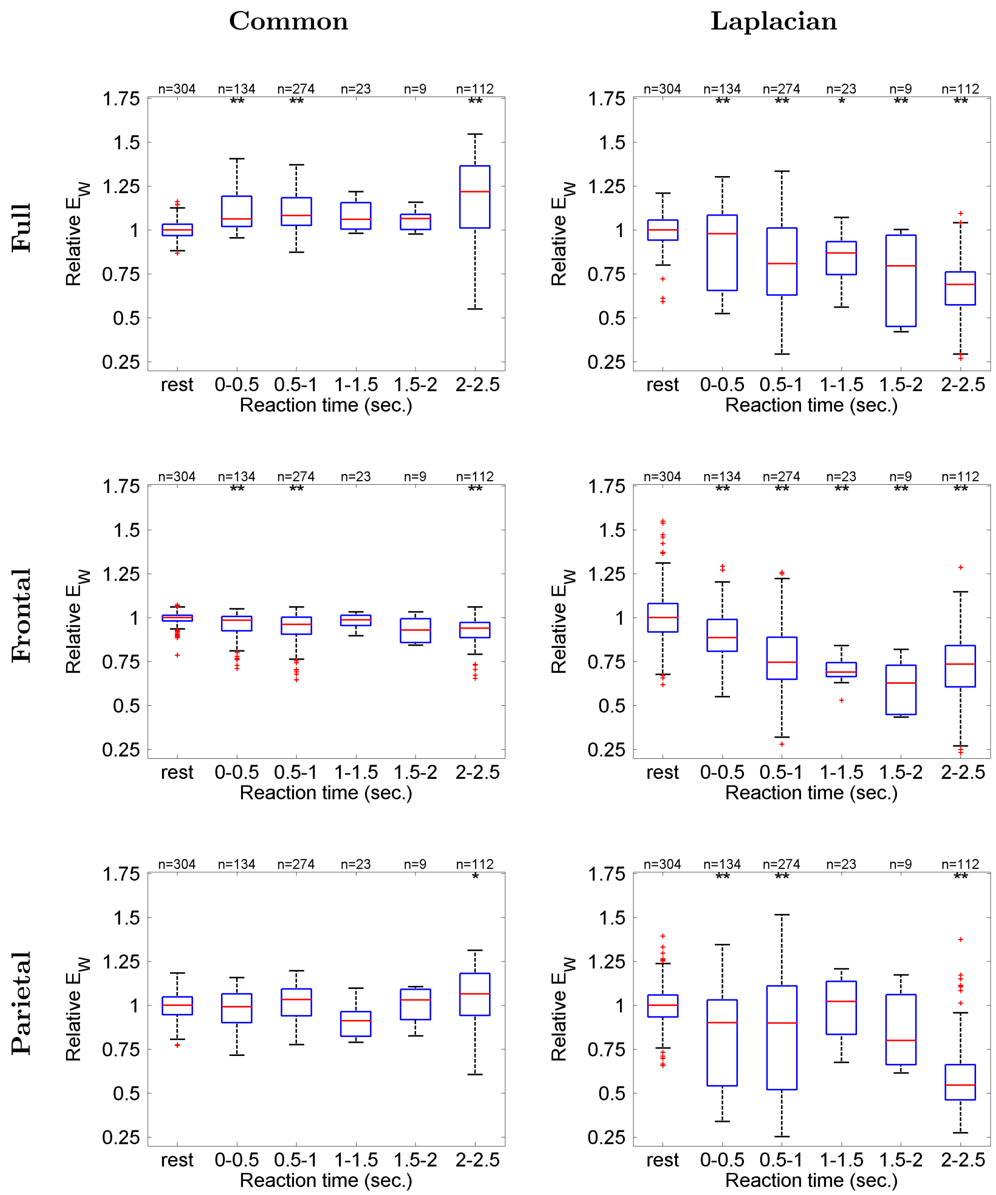

Figure 18. Dependence, for the 60% peak gas group data, of GE-based functional connectivity (E_W) defined relative to the median during rest on aCPT reaction time for full brain (top row), frontal (middle row) and parietal (bottom row) networks, obtained either with common-reference (left column) or Laplacian re-referencing (right column). Box-whisker and multi-comparison test significance marker ($*$,$**$) details are the same as for Figure 5 in the manuscript.

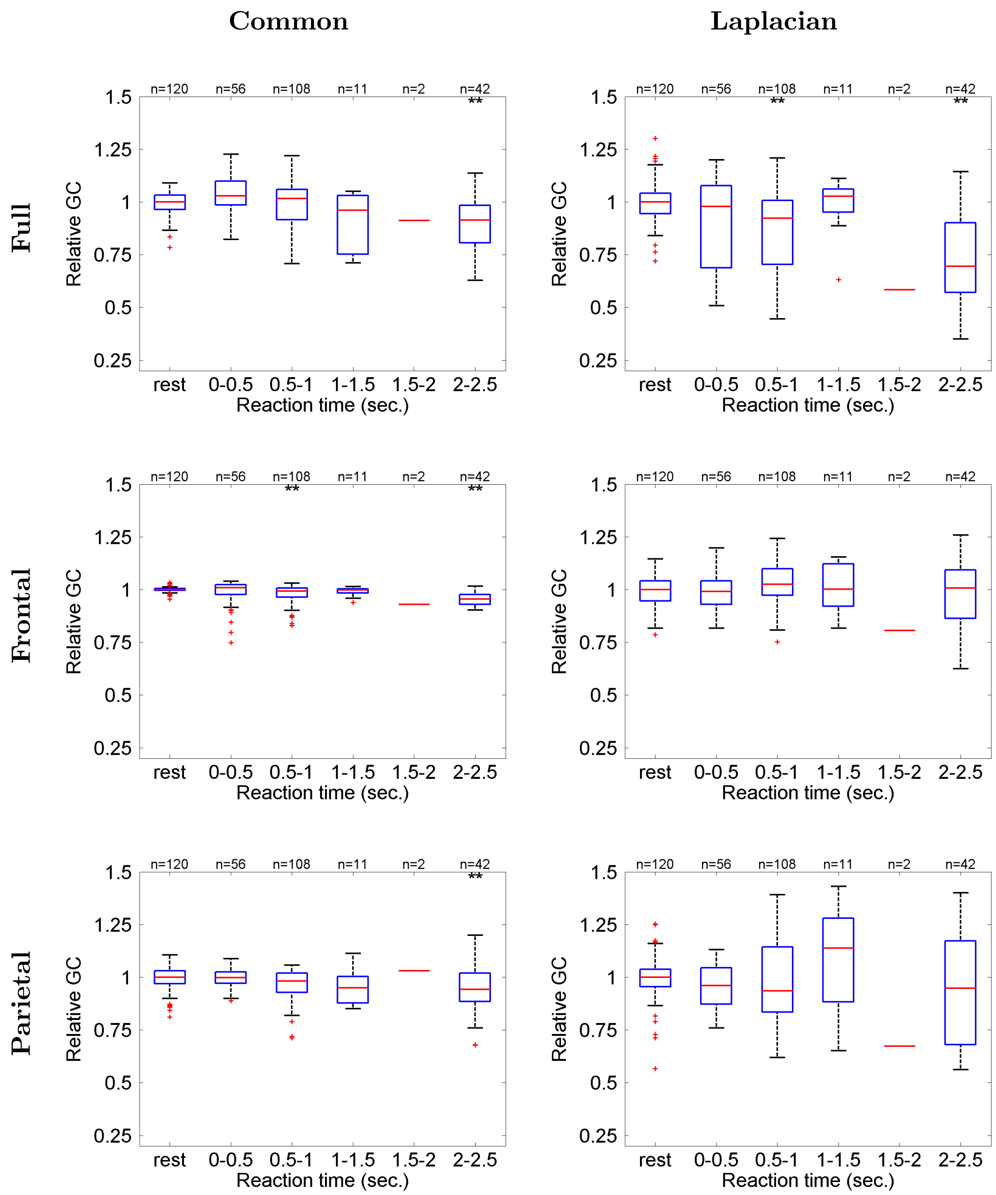

Figure 19. Dependence, for the 60% peak gas group data, of GC-based functional connectivity (C_G) at 11 Hz defined relative to the median during rest on aCPT reaction time for full brain (top row), frontal (middle row) and parietal (bottom row) networks, obtained either with common-reference (left column) or Laplacian re-referencing (right column). Box-whisker and multi-comparison test significance marker ($*$,$**$) details are the same as for Figure 5 in the manuscript.

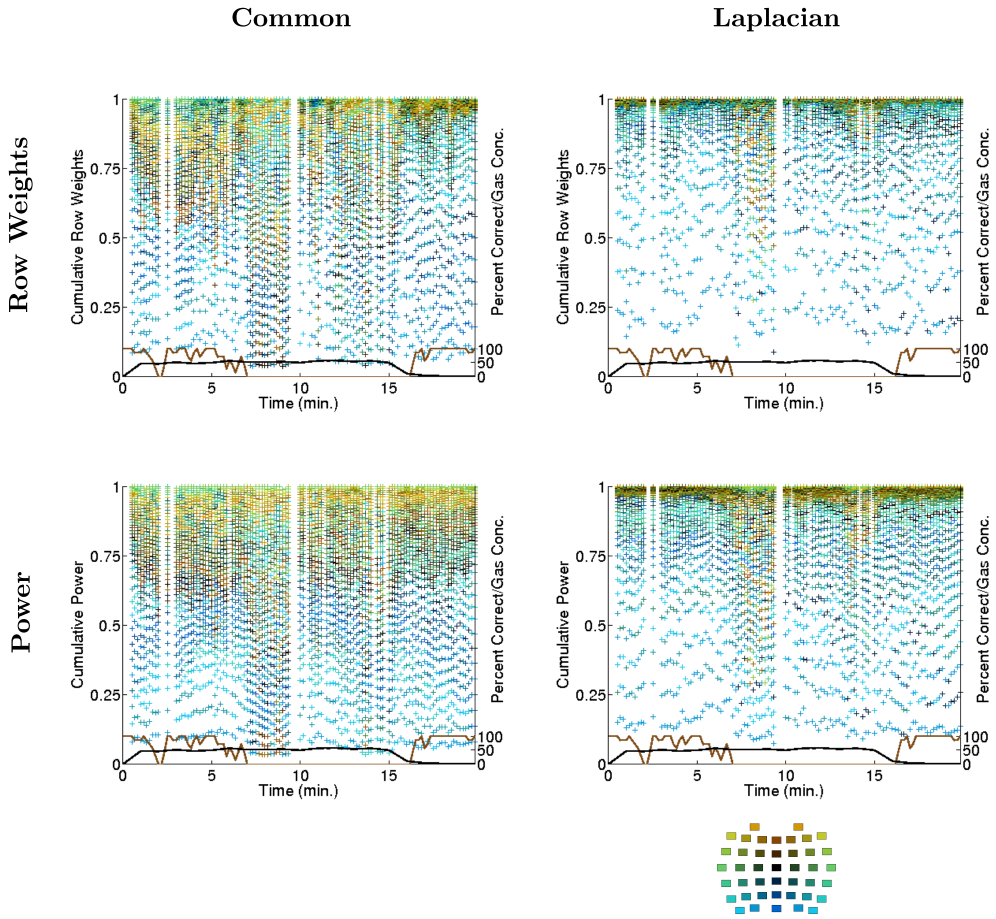

Figure 20. Cumulative row weights (top row) and cumulative power (bottom row) at 11 Hz for the same subject from Figures 1-4 in the 60% peak gas case, with Common-reference (left column) or Laplacian re-referencing (right column). Black curves: end-tidal N_2O gas concentration. Brown curves: auditory task performance. The colormap at the bottom indicates how color codes electrode location with red being anterior and blue being posterior.

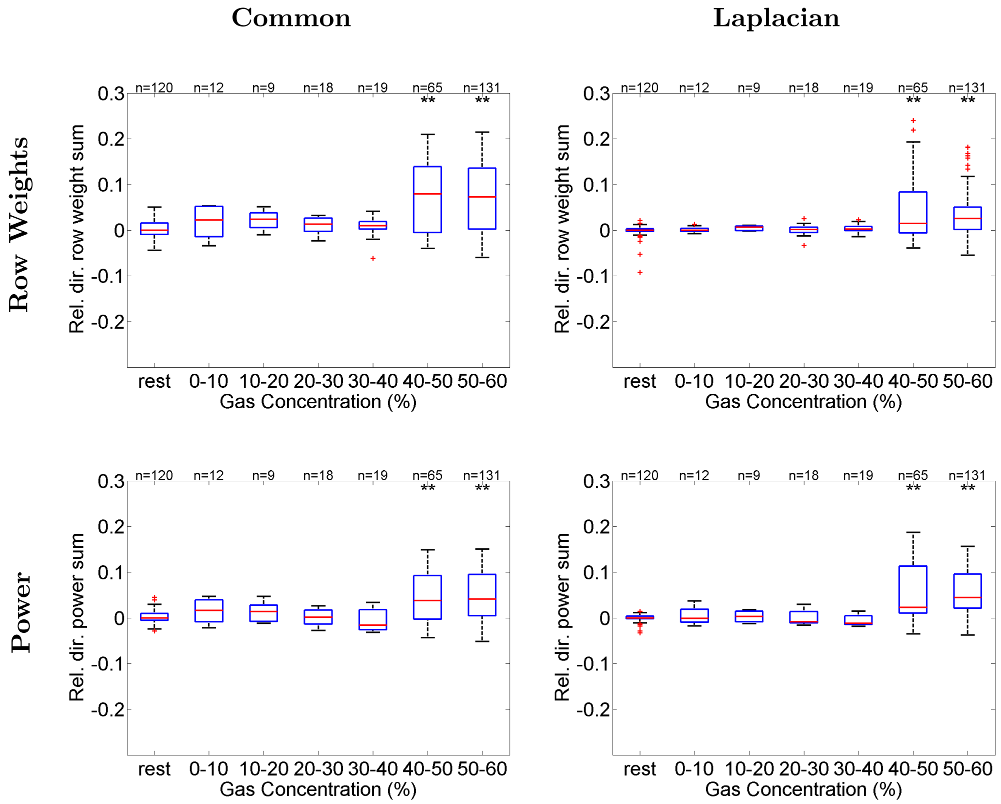

Figure 21. Dependence of dRWS (top row) and dPS (bottom row) at 11 Hz defined relative to the median during rest on N_2O gas concentration for the 60% peak gas group, obtained either with Common-reference (left column) or Laplacian re-referencing (right column). Box-whisker and multi-comparison test significance marker ($*$,$**$) details are the same as for Figure 5 in the manuscript.

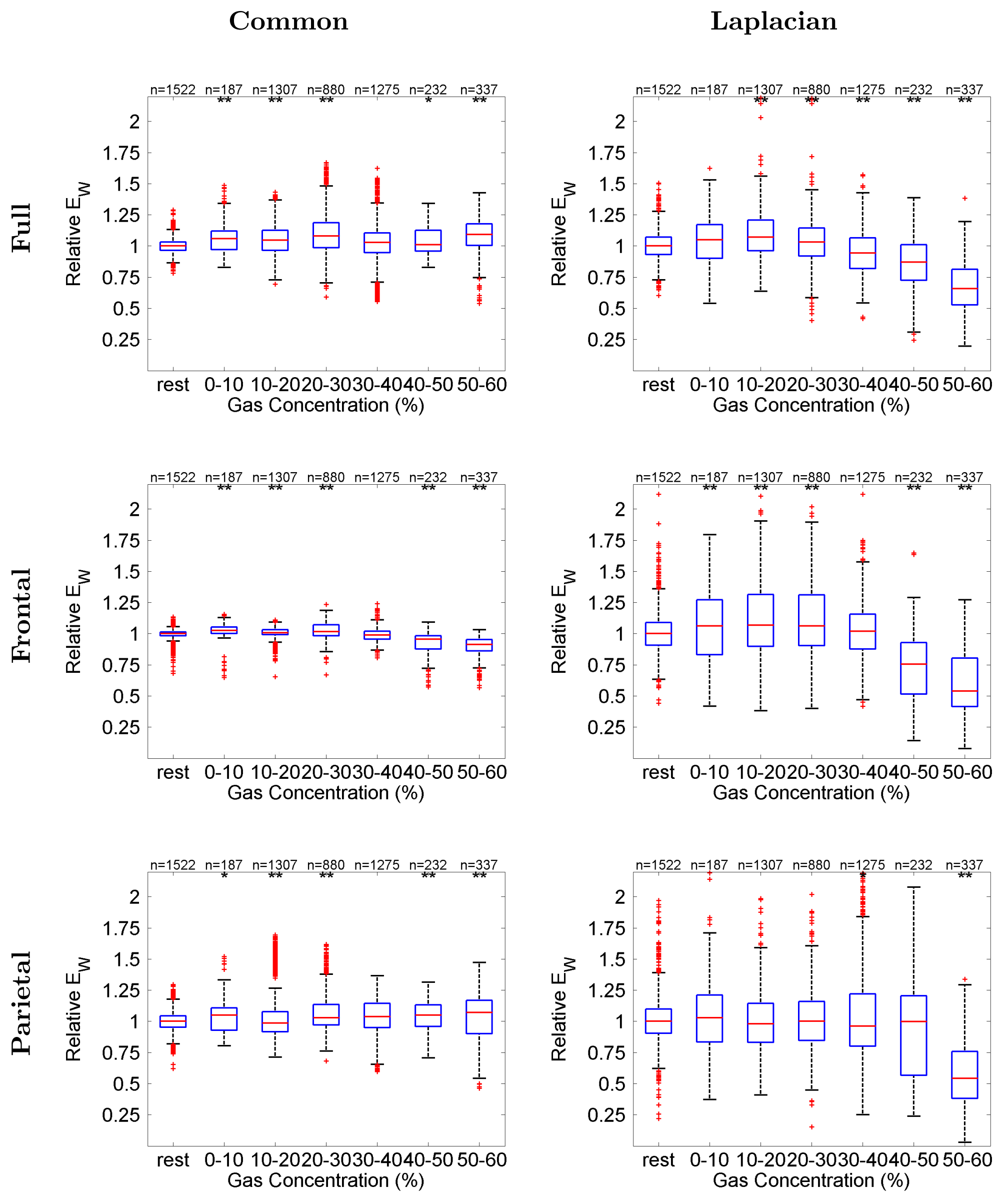

Figure 22. GE analysis with electrodes subsampled to 10-20 montage for the 60% peak gas group: Dependence of GE (E_W) defined relative to the median during rest on N_2O gas concentration for full brain (top row), frontal (middle row) and parietal (bottom row) networks, obtained either with Common-reference (left column) or Laplacian re-referencing (right column). Box-whisker and multi-comparison test significance marker ($*$,$**$) details are the same as for Figure 5 in the manuscript.

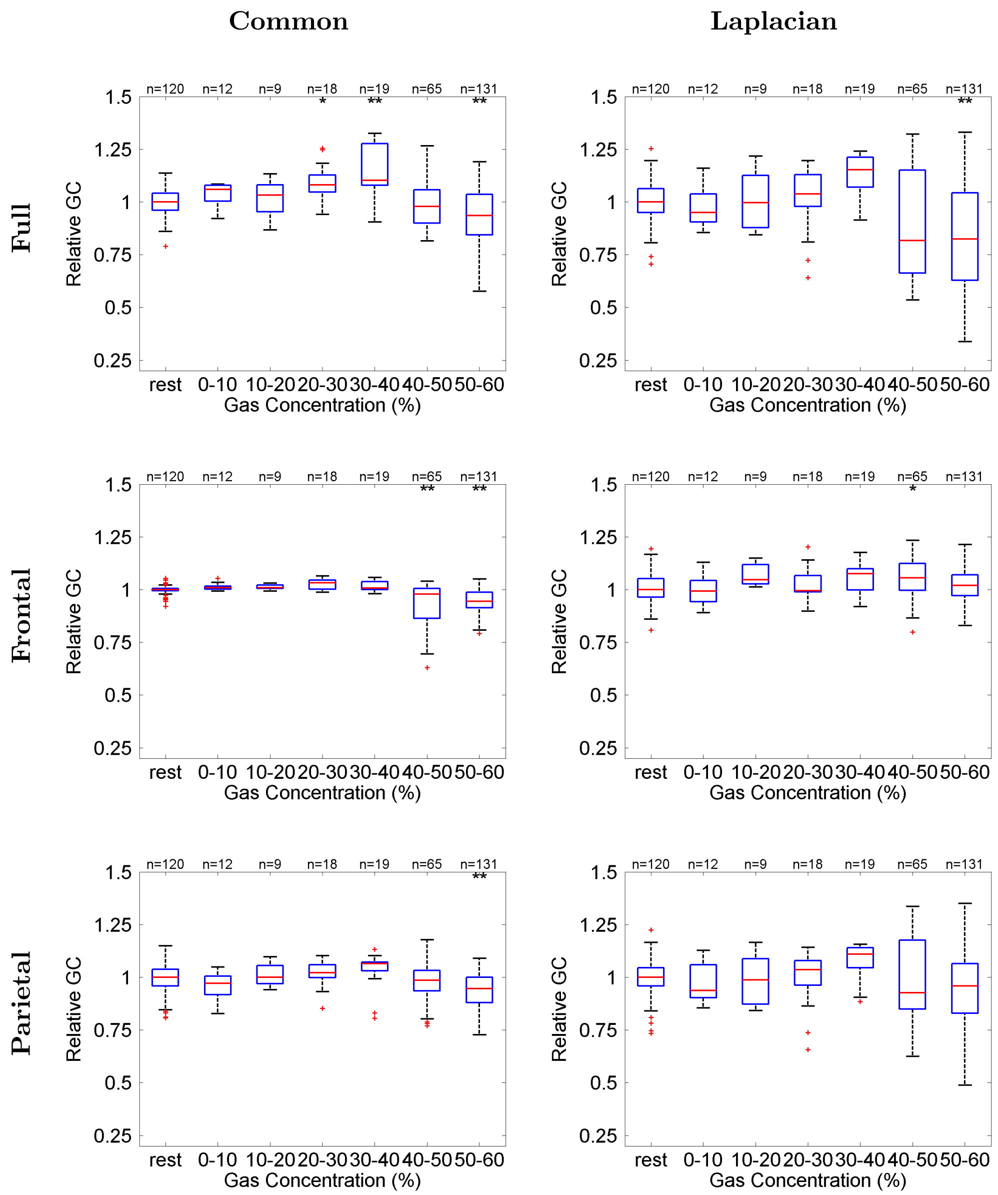

Figure 23. GC analysis with electrodes subsampled to 10-20 montage for the 60% peak gas group: Dependence of GC (C_G) defined relative to the median during rest on N_2O gas concentration for full brain (top row), frontal (middle row) and parietal (bottom row) networks, obtained either with Common-reference (left column) or Laplacian re-referencing (right column). Box-whisker and multi-comparison test significance marker ($*$,$**$) details are the same as for Figure 5 in the manuscript.
